# Genome-Wide Identification, Characterization, and Expression Profiling of the MYC Gene Family in Autotetraploid Alfalfa (*Medicago sativa* L.)

**DOI:** 10.3390/ijms27219964

**Published:** 2026-11-06

**Authors:** Wenjing Ren, Guangzhi Jiang, Jiayin Wang, Huan He, Pingping Du, Hongfei Li, Qinglong Shao, Hongbin Li, Quanliang Xie

**Affiliations:** 1 Key Laboratory of Xinjiang Phytomedicine Resource and Utilization of the Ministry of Education, College of Life Sciences, Shihezi University, Shihezi 832003, China; 17690566315@163.com (W.R.); jiangguangzhi2024@163.com (G.J.); wjyinee@163.com (J.W.); he_huan026@163.com (H.H.); dopingping@126.com (P.D.); 15001612743@163.com (H.L.); 2 Agricultural Science Research Institute of the 3rd Division, Xinjiang Production and Construction Corps, No. 9, Qianhai East Street, Tumushuke 843900, China; 17318743205@163.com

**Keywords:** *Medicago sativa*, *MYC* transcription factor, gene family expansion, salt and drought stress, expression regulation

## Abstract

The *MYC* gene family plays a central regulatory role in plant stress responses; however, its evolutionary dynamics and functional divergence in the polyploid forage legume alfalfa (*Medicago sativa* L.) remain largely unknown. Here, through genome-wide identification and comparative genomic analysis, we systematically identified 38 *MsMYC* genes, which collapsed into 12 ancestral loci based on allele-aware synteny. The high number of sequences was driven mainly by the retention of homoeologous alleles within loci; in addition, one ancient segmental duplication and four tandem duplications contributed to family expansion, whereas strong purifying selection (*Ka*/*Ks* < 1 for the 12 ancient segmental paralog pairs) preserved the core functional architecture. Phylogenetic and structural analyses classified these members into five groups, with inter-group differences in motif composition and exon–intron organization suggesting potential functional divergence. Based on promoter cis-acting element prediction and phylogenetic analysis, we selected eight representative *MsMYC* genes for qRT-PCR analysis, which displayed three representative kinetic behaviors under salt, drought, and hormone treatments within this subset: early-peaking, late-peaking, and opposite-direction responses; subcellular localization analysis of eight representative stress-responsive members supported their nuclear localization, consistent with their roles as transcription factors and with the in silico predictions. Together, these results provide a genomic framework for the temporal and functional differentiation of the *MYC* gene family in polyploid alfalfa. This study provides candidate genes with distinct temporal expression dynamics for the molecular breeding of salt- and drought-tolerant alfalfa.

## 1. Introduction

Alfalfa (*Medicago sativa* L.), owing to its high yield, superior quality, and nitrogen-fixing ability, has been widely recognized as the “king of forage crops”, playing a dominant role in livestock production worldwide and accounting for a substantial share of the global commercial forage market [1]. However, alfalfa productivity is severely constrained by abiotic stresses such as drought and soil salinization [2]. Xinjiang is one of the major regions with concentrated saline-alkali land in China, and soil salinization poses a serious threat to local alfalfa cultivation and production [3]. Salt stress not only inhibits the morphogenesis of alfalfa roots and shoots, but also triggers a series of secondary damage by disrupting intracellular ion homeostasis, ultimately leading to a significant reduction in biomass [4]. Although alfalfa breeding has been carried out both domestically and internationally since the 1930s, compared with that in developed countries, the domestic alfalfa industry in China started late, with weak cultivation techniques and an immature market regulatory system; therefore, the development of elite salt-tolerant varieties remains a critical bottleneck that needs to be addressed urgently.

Transcription factors play pivotal roles in the complex regulatory networks underlying plant responses to salt stress. In recent years, several transcription factor families have been systematically characterized at the genome-wide level in alfalfa, including NAC [5], ARF [6], HSF [7], PLATZ [8], and bZIP [9]. The bHLH (basic helix-loop-helix) family is the second largest class of transcription factors in plants, among which the *MYC* group has attracted considerable attention owing to its involvement in jasmonic acid (JA) signaling. The characteristic domain architecture of MYC proteins consists of an N-terminal MYC_N domain (also known as the JID, Jasmonate Interaction Domain, responsible for JA perception and interaction) and a C-terminal canonical bHLH DNA-binding domain [10]. These proteins bind DNA via the basic region of the bHLH domain and dimerize through the helix-loop-helix region, thereby regulating the expression of downstream target genes [11,12]. Accumulating evidence indicates that *MYC* genes are broadly involved in plant growth and development, secondary metabolism, and stress adaptation. With respect to growth and development, *Arabidopsis AtMYC2*, together with *AtMYC3* and *AtMYC4*, regulates leaf senescence, root elongation, and stamen development [13,14,15]. In *Medicago truncatula*, *MtMYC2* acts as a core transcription factor in the JA signaling pathway: rhizobial inoculation rapidly induces the JA response, and *MtMYC2* regulates the expression of downstream symbiotic genes (e.g., *MtIPD3* and *MtNIN*) to promote infection thread formation and nodule development, while also modulating the expression of nodule-specific defense-related genes (e.g., *MtDNF2* and *MtNAD1*) to suppress excessive host defense, thereby maintaining efficient symbiotic nitrogen fixation [16]. In rice, *OsMYC2* regulates spikelet development through interaction with *OsJAZ1*. With regard to stress responses, the expression of *AtMYC2* is strongly induced by drought and salt stress, and its overexpression enhances osmotic stress tolerance [17,18]; conversely, *AtMYC67* and *AtMYC70* act as negative regulators of freezing tolerance [19]. Furthermore, multiple *MYC* genes have been confirmed as core regulatory nodes in the JA signaling pathway and play dual roles in resistance to biotic stress [20,21,22]. Although the *MYC* gene family has been relatively well characterized in *Arabidopsis*, rice, and *M. truncatula*, the identification, evolutionary dynamics, and abiotic stress response mechanisms of *MYC* gene family members in polyploid alfalfa (*Medicago sativa* L.), an important forage crop, remain poorly understood. Considering that tetraploid alfalfa is an autotetraploid and may show partial diploidization at some loci, the patterns of gene family expansion and functional redundancy are likely more complex than those in diploid species [23,24]. For tetraploid alfalfa, the number, genomic distribution, and structural diversity of *MYC* gene family members remain unclear, which directly limits our understanding of functional retention and divergence during the evolution of this family. Moreover, whether the expression regulatory networks of *MYC* genes undergo reprogramming under salt and drought stress, and whether different members exhibit stress-response biases currently lack systematic evidence. Therefore, systematically dissecting the structural characteristics and stress response patterns of the *MYC* gene family in alfalfa is of great significance for elucidating the molecular mechanisms underlying stress adaptation in polyploid plants.

In this study, we performed the first systematic identification and characterization of the *MYC* gene family at the whole-genome level in alfalfa. Through phylogenetic analysis, collinearity analysis, and promoter prediction, we revealed the group classification, duplication events, and promoter cis-acting element composition of this family. To investigate the stress response mechanisms of this family, the autotetraploid cultivar ‘Xinjiang Daye’ was selected as the experimental material because it is a major local cultivar adapted to the saline-alkali soils of Xinjiang, and its recently published allele-aware genome assembly provides a reliable reference for gene family analysis. Leaves were chosen as the target tissue because they are the primary organs for photosynthesis and are highly sensitive to abiotic stress. Since MYC transcription factors are core regulators of the JA pathway, and JA often interacts antagonistically or synergistically with salicylic acid (SA), exogenous MeJA and SA treatments were applied to explore their regulatory roles. Furthermore, NaCl and drought treatments were also applied. By integrating promoter cis-element analysis and qRT-PCR analysis, we identified candidate genes responsive to salt, drought, and hormone treatments at short-term (0–24 h) and long-term (1–7 d) stages, with sampling time points designed according to previous stress response studies, and examined their subcellular localization. This study not only provides new insights into the evolution and function of *MYC* genes in polyploid crops, but also offers candidate genetic resources for molecular breeding of salt-tolerant alfalfa.

## 2. Results

### 2.1. Identification and Classification of the MsMYC Gene Family

**Figure 1:**
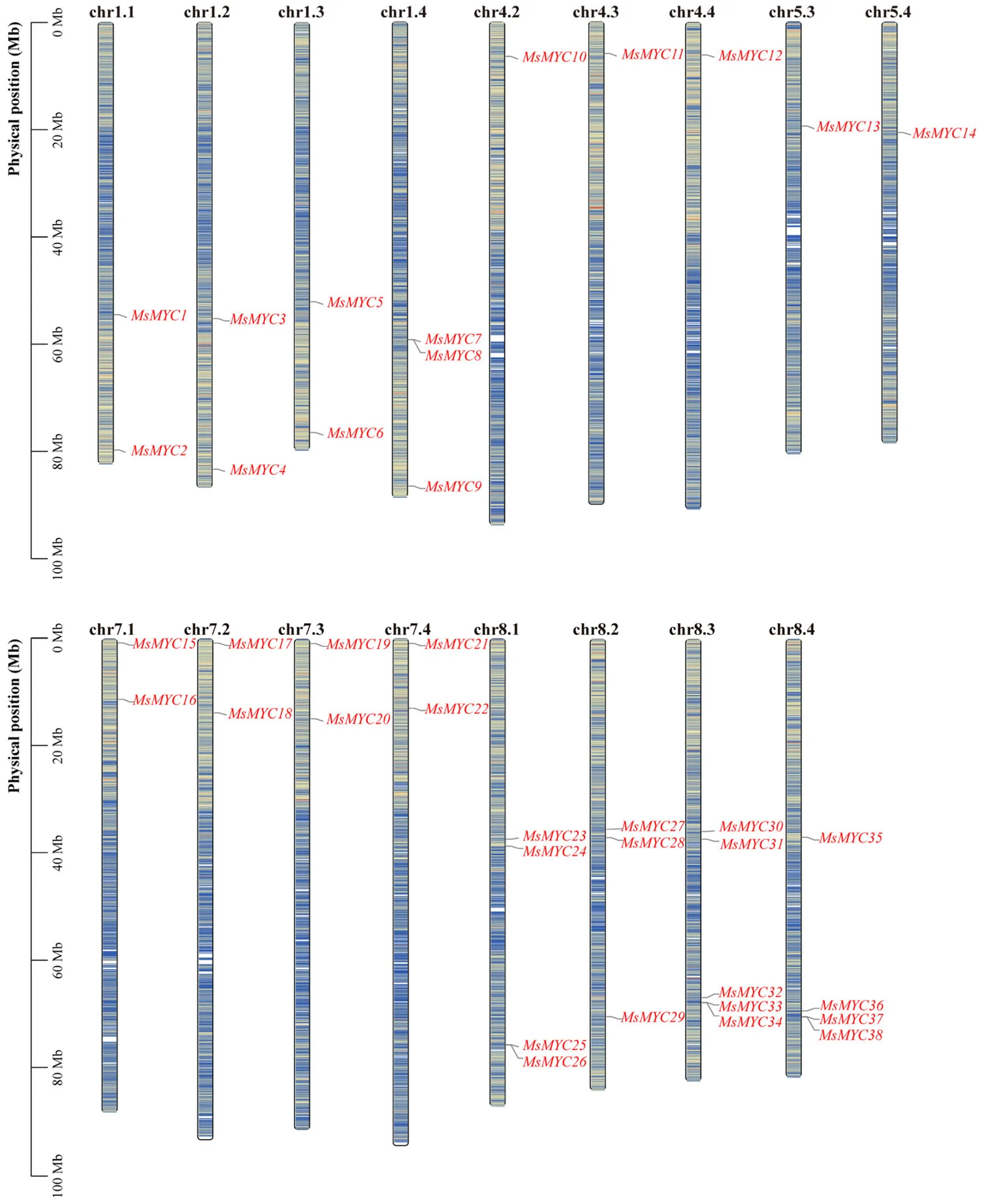
Schematic representation of chromosomal distribution of *MsMYC* genes in alfalfa. Vertical bars represent the 32 pseudochromosomes of the autotetraploid alfalfa genome, labeled according to the allele-aware assembly (chr1.1–chr8.4) and grouped by homologous group; the scale bar on the left indicates chromosome length in Mb, and bar width is proportional to chromosome length. Chromosome numbers are shown in black at the top, and *MsMYC* genes are marked in dark red.

Using *Arabidopsis* MYC protein sequences as queries, BLASTP searches were performed against the protein sequences of the alfalfa cultivar ‘Xinjiang Daye’ genome. Meanwhile, hidden Markov model (HMM) profiles of the conserved MYC domains (PF14215 and PF00010) were used for genome-wide searches via HMMER. After confirmation that the candidate sequences obtained from both strategies contained both the MYC_N and bHLH domains using the NCBI CDD, InterPro, and SMART databases, a total of 38 *MsMYC* genes were ultimately identified. The genome assembly used here is the allele-aware chromosome-level assembly of the autotetraploid cultivar ‘Xinjiang Daye’, which comprises 32 pseudochromosomes. The 38 *MsMYC* genes were unevenly distributed across 17 of the 32 pseudochromosomes (alfalfa is an autotetraploid, 2*n* = 4*x* = 32, and the remaining 15 chromosomes contained no *MsMYC* genes), with clear chromosomal enrichment and clustering (Table S1). The genes were highly enriched on chromosomes corresponding to homologous group 8 (based on the homologous group 1–8 numbering system used in this study), where 16 *MsMYC* genes were located (42.1% of the family), whereas homologous groups 5 and 6 harbored the fewest members (2 and 1 genes, respectively). According to their physical positions on the chromosomes, these genes were subsequently designated *MsMYC1* to *MsMYC38* (Figure 1, Table S1). Duplication events were then formally classified. Tandem duplicates were defined as family members located on the same chromosome within 100 kb of each other and separated by no more than 10 intervening genes; four tandem pairs were identified (*MsMYC7*/*MsMYC8*, *MsMYC25*/*MsMYC26*, *MsMYC33*/*MsMYC34* and *MsMYC37*/*MsMYC38*), involving eight genes (21.1% of the family; intergenic distances 27.7–58.3 kb; Table S1). Segmental/WGD duplicates detected by intra-species collinearity analysis comprised 46 gene pairs involving 27 genes (71.1%); these were further resolved by synonymous substitution rate (*Ks*) analysis into recent whole-genome-duplication (WGD)-derived homoeologous pairs and older segmental duplicates (Section 2.4). The remaining three genes (*MsMYC10*, *MsMYC13* and *MsMYC14*) had no detectable duplication partner. The assignment of each locus to one of the four homologous chromosome sets was based on the allele-aware annotation of the reference genome (Table S1).

To avoid counting homoeologous alleles as independent paralogs, the 38 *MsMYC* genes were collapsed into ancestral loci based on the allele-aware assembly and four-way homoeologous synteny. This collapsed the 38 genes into 12 ancestral loci (Table S7). Of these, five loci retained all four homoeologous copies, whereas the remaining loci retained two or three copies, indicating partial homoeolog loss after autotetraploidization.

Physicochemical property analysis (Table S1) showed that the amino acid length of MsMYC proteins ranged from 419 aa (MsMYC28) to 678 aa (MsMYC14), and the molecular weight ranged from 47.8 kDa (MsMYC28) to 74.6 kDa (MsMYC14). The theoretical isoelectric point (pI) ranged from 4.84 (MsMYC7) to 8.81 (MsMYC35), among which 34 members (approximately 89.5%) had pI values below 7.0, indicating that this family is predominantly composed of acidic proteins. The instability index ranged from 35.90 to 63.21, with 26 members (68.4%) exhibiting values greater than 40. The aliphatic index ranged from 67.43 (MsMYC14) to 89.07 (MsMYC28). The grand average of hydropathy (GRAVY) values of all members was negative, ranging from −0.764 (MsMYC6) to −0.389 (MsMYC24), indicating that all MsMYC proteins are hydrophilic, which is consistent with the function of transcription factors in the intracellular aqueous environment.

Subcellular localization prediction showed that all MsMYC proteins were localized in the nucleus (Table S2). Secondary structure analysis (Table S2) indicated that in all MsMYC proteins, random coils (52.72–73.12%) and α-helices (22.73–36.82%) were the predominant structural elements, while the content of extended strands was relatively low (4.15–10.78%), and none of the members contained β-turns. This structural feature is compatible with the inherent conformational flexibility of transcriptional regulatory factors, which is conducive to mediating protein–protein and protein–DNA interactions.

### 2.2. Phylogenetic Analysis of MsMYC Proteins

**Figure 2:**
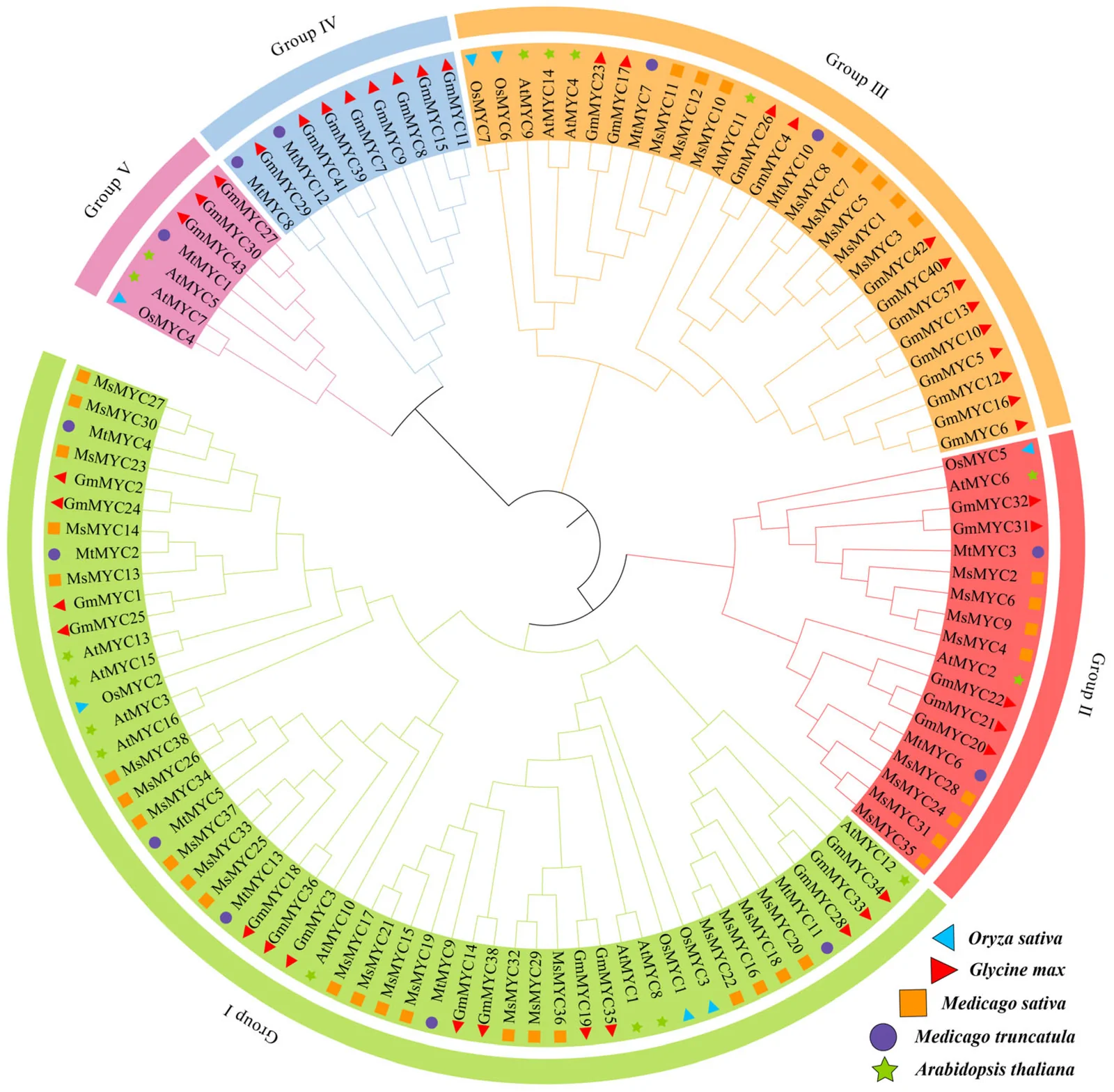
Phylogenetic tree of MYC proteins from alfalfa and other plant species. The tree was constructed using the neighbor-joining method, including sequences from *Medicago sativa* (MsMYCs), *Arabidopsis thaliana* (AtMYCs), *Medicago truncatula* (MtMYCs), *Glycine max* (GmMYCs) and *Oryza sativa* (OsMYCs). The five subfamilies (Groups I–V) are indicated by shaded sectors and species are distinguished by branch color (see inset legend).

To investigate the evolutionary relationships among MsMYC proteins, a dataset comprising 117 MYC protein sequences from five species was constructed. Among them, sequences from *Arabidopsis thaliana*, *Medicago truncatula*, and *Glycine max* were obtained from previously published studies, whereas sequences from *Medicago sativa* and *Oryza sativa* were identified from their respective genomes through local BLASTP and HMMER searches. Based on these sequences, phylogenetic trees were constructed using the neighbor-joining (NJ; Figure 2) and maximum likelihood (ML; Figure S4) methods. The MYC proteins were classified into five subfamilies, designated Groups I to V. Among them, MsMYC13, MsMYC14, MsMYC23, MsMYC25–MsMYC27, MsMYC30, MsMYC33, MsMYC34, MsMYC37, and MsMYC38 belonged to Group I; MsMYC15–22, 29, 32, and MsMYC36 belonged to Group II; MsMYC1, 3, 5, 7, 8, and MsMYC10–12 belonged to Group III; and MsMYC2, 4, 6, 9, 24, 28, 31, and MsMYC35 belonged to Group IV. Notably, no alfalfa MYC members were identified in Group V, which was composed exclusively of MYC sequences from other species, including *Arabidopsis thaliana*, *Medicago truncatula*, *Glycine max*, and *Oryza sativa*. This result could reflect evolutionary loss of the Group in alfalfa, or substantial sequence divergence that prevented annotation of the corresponding loci as typical MYC members. To test whether this reflects gene loss or incomplete annotation, Group V MYC protein sequences were used as queries in tBLASTn searches against the complete alfalfa assembly, including unanchored scaffolds and unplaced contigs (Section 4.2). Multiple significant hits were recovered. After re-examination against the original HMMER/Pfam/CDD criteria, these loci were not retained as *MsMYC* members because their MYC_N domain was incomplete or supported only at the superfamily level, their bHLH region lacked a specific MYC-type hit, or they belonged to the LHW-like subclass (cd18915; IPR043561) rather than the canonical AtMYC2-like subclass (cd11449) (Table S6). These results indicate that Group V-related MYC sequences are present in the alfalfa genome, which is more consistent with incomplete annotation or extensive sequence divergence than with genuine gene loss. Nevertheless, including these subfamilies in the phylogenetic analysis contributes to a more comprehensive understanding of the evolutionary divergence patterns of MYC proteins across different plant lineages.

### 2.3. Conserved Motif, Domain, and Gene Structure Analysis of the MsMYC Gene Family

**Figure 3:**
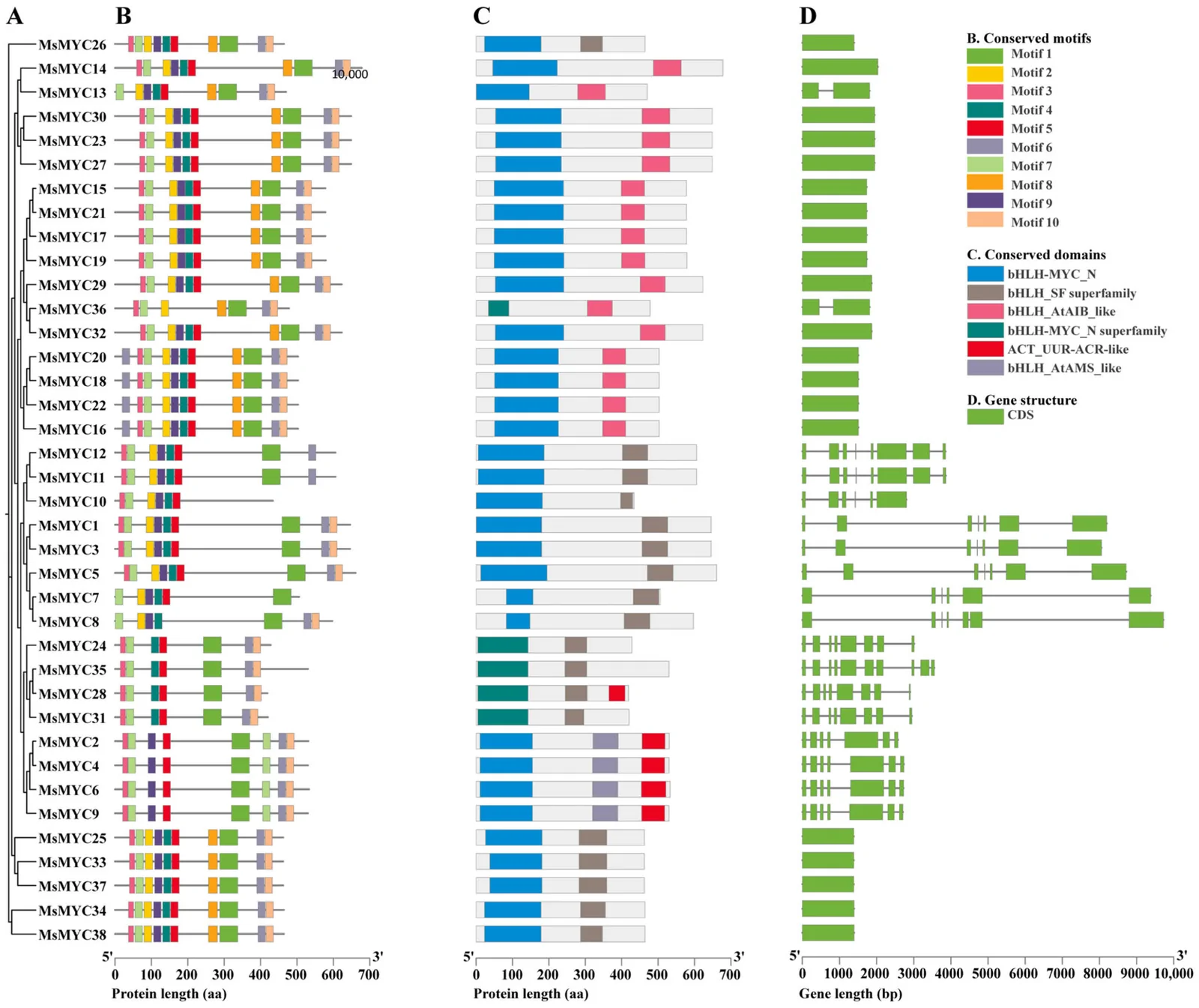
Phylogenetic relationships, conserved motifs, conserved domains, and gene structures of MsMYC proteins. Panels are arranged from left to right in the same order as they are described in the text: (**A**) phylogenetic tree of MsMYC proteins; (**B**) distribution of the 10 conserved motifs, with the *x*-axis indicating the protein length (aa); (**C**) distribution of conserved domains, with the *x*-axis indicating the protein length (aa); (**D**) gene structures, in which green rectangles represent exons (CDS), and black lines represent introns and untranslated regions (UTRs), with the *x*-axis indicating the genomic sequence length (bp). The legends for each panel are separately grouped on the right side.

To further investigate the evolutionary relationships within the *MsMYC* gene family, a comprehensive visualization analysis of conserved motifs, domains, and gene structures was performed for its 38 members (Figure 3). Motif analysis identified a total of 10 conserved motifs (Figure 3). All MsMYC proteins contained Motif 7, and the vast majority of MsMYC proteins contained Motifs 3 and 6, indicating that these motifs are highly conserved within the gene family and are likely of critical functional importance. Furthermore, significant differences in motif composition were observed among different subfamilies. MsMYC proteins within the same Group exhibited similar motif numbers and types. For example, all members of Group III possessed Motifs 1, 3, 5, 6, 7, and 10.

To investigate the structural characteristics of MsMYC proteins, their conserved domains were analyzed using the Pfam and SMART databases. The results showed that all MsMYC proteins contained the characteristic MYC domain. Gene structure analysis revealed that the number of exons in *MsMYC* genes varied considerably, ranging from 1 to 10. Among them, the gene *MsMYC35* contained the highest number of exons (10). In contrast, all members of Groups I and II, except for *MsMYC13* and *MsMYC36*, contained only one exon (Figure 3).

These results indicate that each Group has a distinct motif composition and gene structure, and that members within each Group share similar conserved motifs. Although members of the *MYC* gene family remained relatively conserved in sequence features, the differences in domain composition and gene structure among subfamilies suggested that a certain degree of functional divergence may have occurred, which awaits further verification through expression and functional analyses.

### 2.4. Gene Duplication Events and Collinearity Analysis of the MsMYC Gene Family

**Figure 4:**
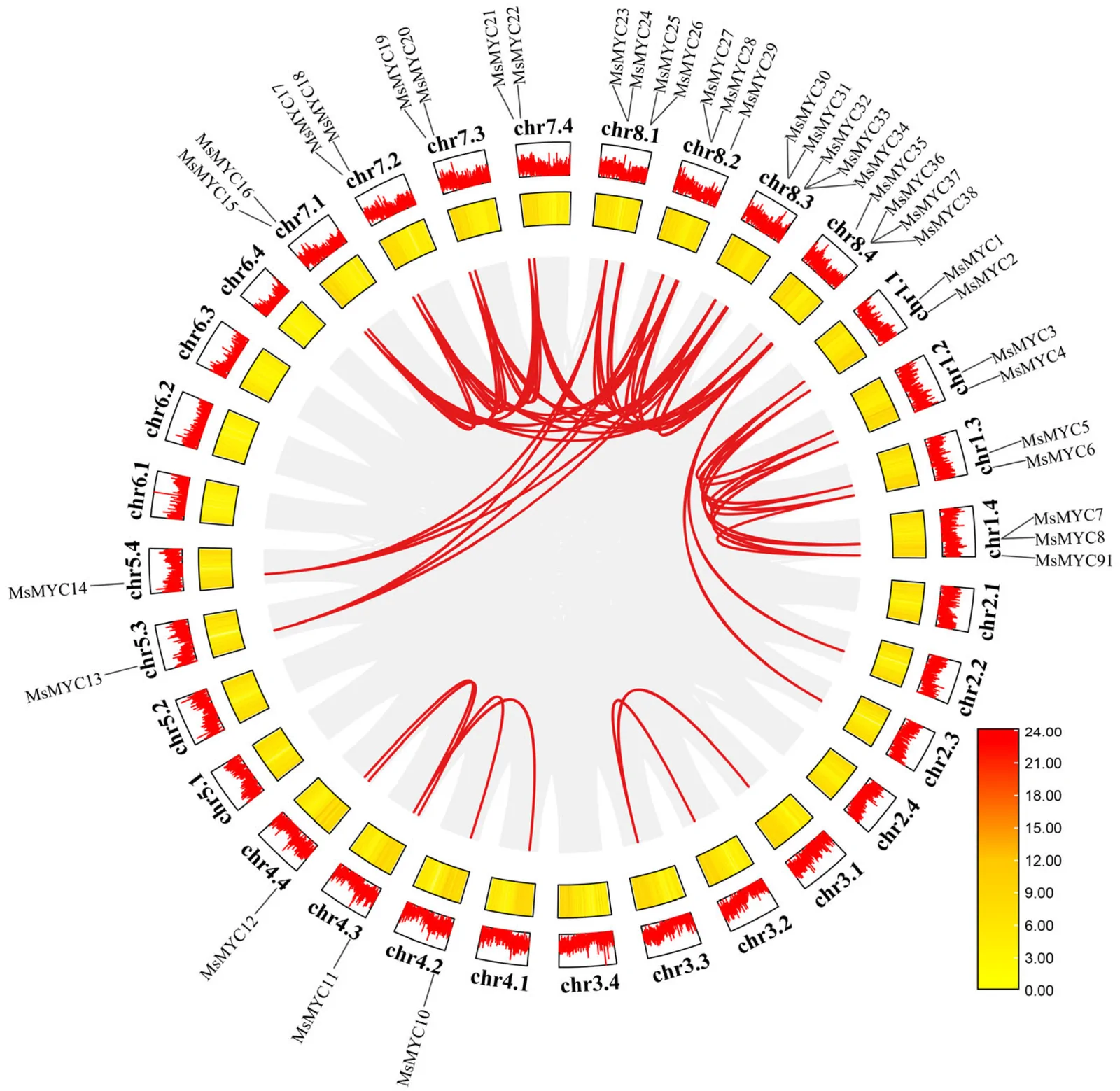
Circular visualization of collinearity among *MsMYC* genes. Chromosomal positions of *MsMYC* genes are shown on the outer ring. The yellow and red outer circles represent gene density. segmental/WGD duplicated *MsMYC* gene pairs (n = 46) are connected by red curves; genome-wide collinear blocks are shown in grey in the background. Gene density (yellow track) and the tandem-duplication clusters are annotated on the outer ring.

**Figure 5:**
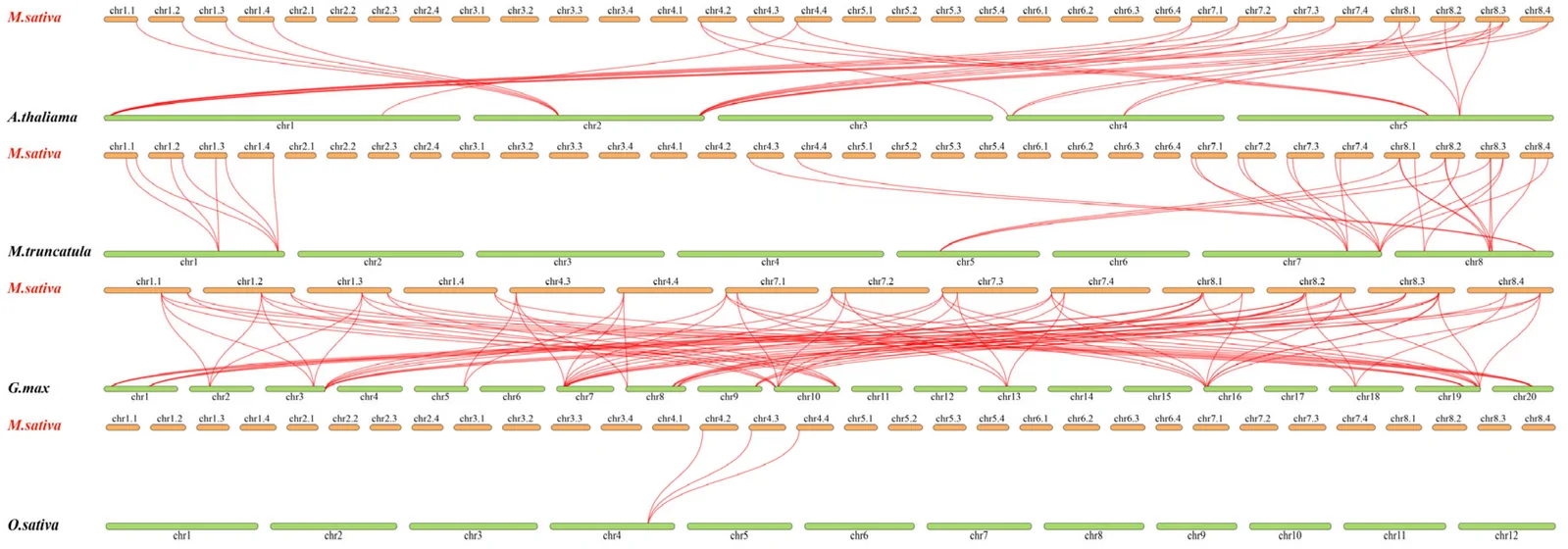
Syntenic relationships of *MYC* members between *Medicago sativa* and four other plant species. Red lines connect homologous *MsMYC* gene pairs.

To further investigate potential gene duplication events among *MsMYC* genes, we performed intra-species collinearity analysis based on the genome database of the ‘Xinjiang Daye’ cultivar (Figure 4). The results showed that the *MsMYC* genes were involved in 46 WGD/segmental duplicated gene pairs identified from intra-species collinearity analysis, comprising 27 family members (71.1%). Because intra-species collinearity alone cannot separate homoeologous pairs produced by the recent autotetraploidization from older segmental duplications, the 46 pairs were further classified using the distribution of synonymous substitution rates (*Ks*) together with the depth of syntenic support (Figure 4, Table S3). After collapsing to loci, these 46 pairs were reclassified into two categories. First, 34 pairs were classified as homoeologous allele pairs within the same locus (28 pairs with *Ks* ≤ 0.05, plus six pairs among *MsMYC24*, *MsMYC28*, *MsMYC31* and *MsMYC35* with *Ks* 0.063–0.116); these represent allelic variation, not independent paralogs. Second, 12 pairs (*Ks* 0.74–0.79) were classified as ancient segmental paralogs. In addition, four tandem paralog pairs were identified. Thus, true paralogous expansion of the MsMYC family is limited to one ancient segmental duplication and four tandem duplications, whereas the recent WGD-derived pairs represent homoeologous alleles retained within ancestral loci. Two pairs (*MsMYC17*/*MsMYC21* and *MsMYC18*/*MsMYC20*) had *Ka* = *Ks* = 0, indicating essentially identical sequences and therefore extremely recent allelic variation. These two pairs were excluded from *Ka*/*Ks* analysis because *Ka*/*Ks* is mathematically undefined. *Ka/Ks* analysis showed that the 12 ancient segmental paralog pairs were under strong purifying selection (Fisher’s exact test, *p* < 0.0001; Table S3). Furthermore, to explore the phylogenetic relationships of the *MYC* gene family among different plant species and to elucidate the contribution of polyploidization to the retention of MYC copies, we performed inter-species collinearity analysis among *Medicago sativa*, *Arabidopsis thaliana*, *Medicago truncatula*, *Glycine max*, and *Oryza sativa* (Figure 5). The results showed that alfalfa (*Medicago sativa*) shared 26, 30, 87, and 3 homologous gene pairs with *Arabidopsis thaliana*, *Medicago truncatula*, *Glycine max*, and *Oryza sativa*, respectively (Figure 5, Table S4). The number of homologous gene pairs between alfalfa and legume species was much higher than that between alfalfa and non-legume species, consistent with a closer phylogenetic relationship between alfalfa and other legumes and with the high conservation of *MYC* genes in this clade. To assess gene retention after polyploidization, we compared the number of *MsMYC* genes that retained a syntenic counterpart in each species with the total number of family members (retention rate): 27 of 38 alfalfa genes (71.1%) had a syntenic counterpart in the diploid relative *M. truncatula* and the reciprocal retention rate in *M. truncatula* was 9 of 13 genes (69.2%); the corresponding values were 16 of 38 (42.1%) for *A. thaliana*, 30 of 38 (78.9%) for *G. max* and 3 of 38 (7.9%) for *O. sativa*. Retention relative to *M. truncatula* was therefore high, and a single *M. truncatula MYC* gene was frequently matched by several alfalfa loci (up to six), indicating that multiple homoeologous copies were retained rather than one copy being systematically lost. Whether the MYC family shows biased fractionation, that is, preferential loss of copies derived from one of the four homologous chromosomes within each homologous group, remains to be determined.

### 2.5. Promoter Cis-Acting Element Analysis of the MsMYC Gene Family

**Figure 6:**
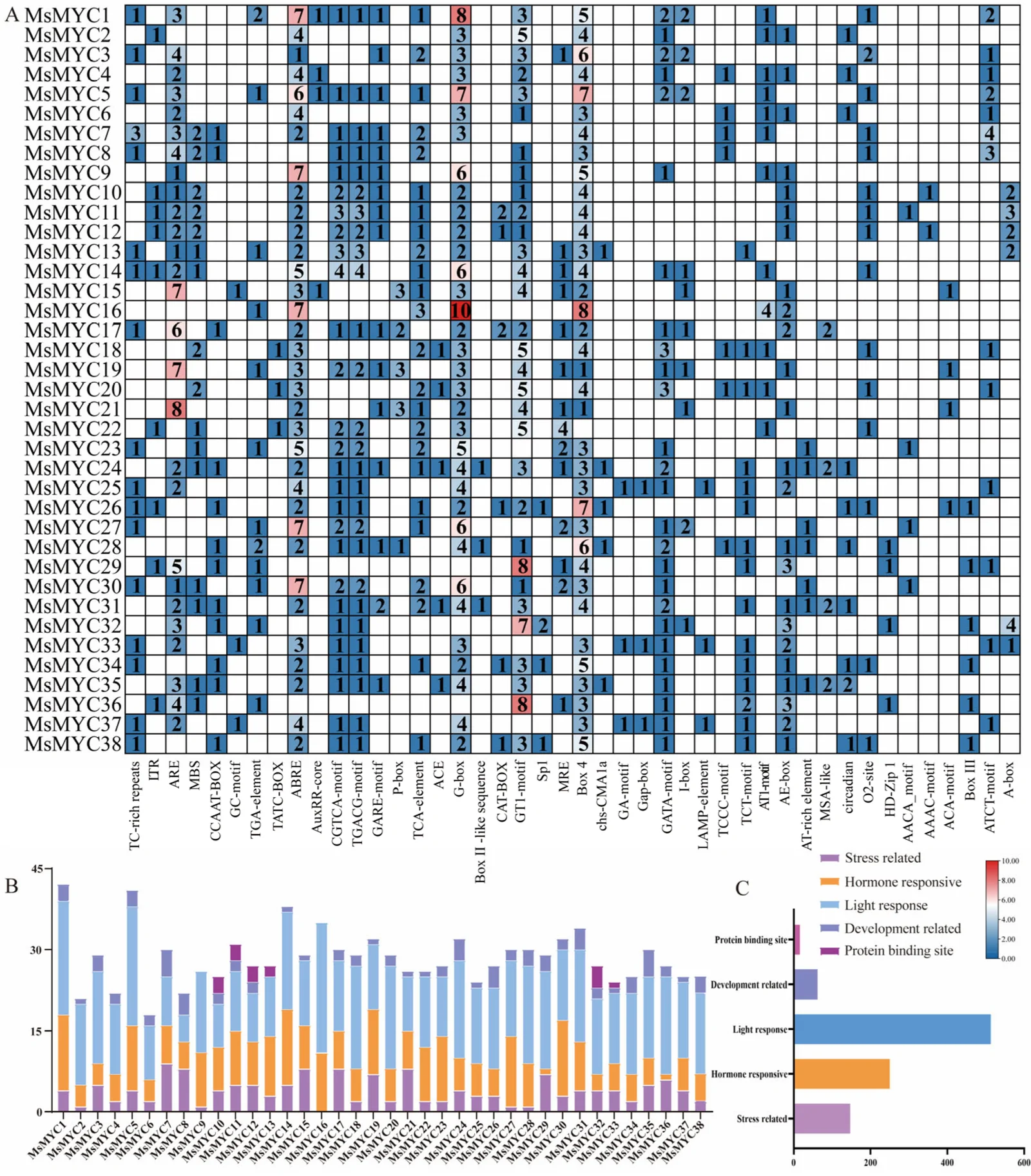
Statistics and classification of cis-acting elements in the *MsMYC* gene family. (**A**) Heatmap of cis-acting element classification, divided into five categories. Numbers in the heatmap represent the counts of cis-acting elements. (**B**) Statistics of cis-acting elements in each *MsMYC* gene. Different colors in the stacked bar chart represent different categories. (**C**) Classification of all cis-acting elements in the *MsMYC* gene family. Different colors represent different categories.

To predict the functions and regulatory mechanisms of *MsMYC* genes, PlantCARE was used to identify and analyze potential cis-acting elements in the 2 kb sequences upstream of the start codon of these genes. As shown in Figure 6, a total of 44 types of cis-acting elements were identified in the promoter regions of *MsMYC* genes. These included 18 types related to light response; 6 types related to protein-binding sites, including specific protein-binding sites, transcription factor binding sites such as NF-Y and SP1, and matrix attachment regions that bind nuclear matrix proteins. In addition, 7 types were associated with development, including elements involved in meristem expression, palisade mesophyll cell differentiation, circadian rhythm control, cell cycle regulation, endosperm-specific negative regulation, and prolamin metabolism; 9 types were associated with hormone responses, including elements responsive to abscisic acid, auxin, methyl jasmonate, gibberellin, and salicylic acid; and 4 types were associated with stress responses, including low-temperature-, drought-, and anaerobic-responsive elements. In the *MsMYC* gene family, light-responsive elements were the most abundant (513 elements), followed by hormone-responsive (250) and stress-responsive (147) elements. Among the family members, *MsMYC1* contained the highest number of cis-acting elements (42) and *MsMYC6* the lowest (18). TATA-box and CAAT-box were excluded from the functional interpretation, and the subsequent analysis focused on hormone- and stress-responsive elements. At least one of the five types of hormone- and stress-responsive elements (MeJA-responsive CGTCA-motif and TGACG-motif, ABA-responsive ABRE, SA-responsive TCA-element, drought-responsive MBS, and anaerobic-responsive ARE) was detected in 100% of the *MsMYC* promoters, with a mean of 6.7 elements per gene (Figure 6A; Figure S4). The promoters of *MsMYC12* and *MsMYC23* contained four MeJA-responsive elements each, and two and five ABA-responsive elements, respectively; in contrast, no MeJA- or ABA-responsive element was detected in the promoter of *MsMYC36*. Across the family, the number of hormone- and stress-responsive elements varied considerably among members.

### 2.6. Expression Pattern Analysis of MsMYC Genes Under Different Abiotic Stress Treatments

To investigate the responses of *MsMYC* gene family members to MeJA, SA, NaCl, and drought treatments, eight members (*MsMYC11*, *MsMYC12*, *MsMYC14*, *MsMYC23*, *MsMYC27*, *MsMYC30*, *MsMYC32*, and *MsMYC36*) were selected for qRT-PCR analysis. Selection followed a three-criterion procedure: (i) representation of different phylogenetic groups based on the phylogenetic tree (Figure 2); (ii) presence of at least one MeJA-, ABA-, or drought-responsive cis-element in the 2 kb promoter region (Figure 6 and Figure S4); and (iii) retention of complete MYC_N and bHLH domains. The experimental design for the qRT-PCR analysis, including treatment conditions, time points, and biological replicates, is described in Section 4.6.

In this study, “stress-responsive *MsMYC* genes” refers to the eight genes that showed significant expression changes (*p* < 0.05, one-way ANOVA followed by Duncan’s test) relative to the untreated control in at least one of the NaCl, drought, MeJA, or SA treatments.

**Figure 7:**
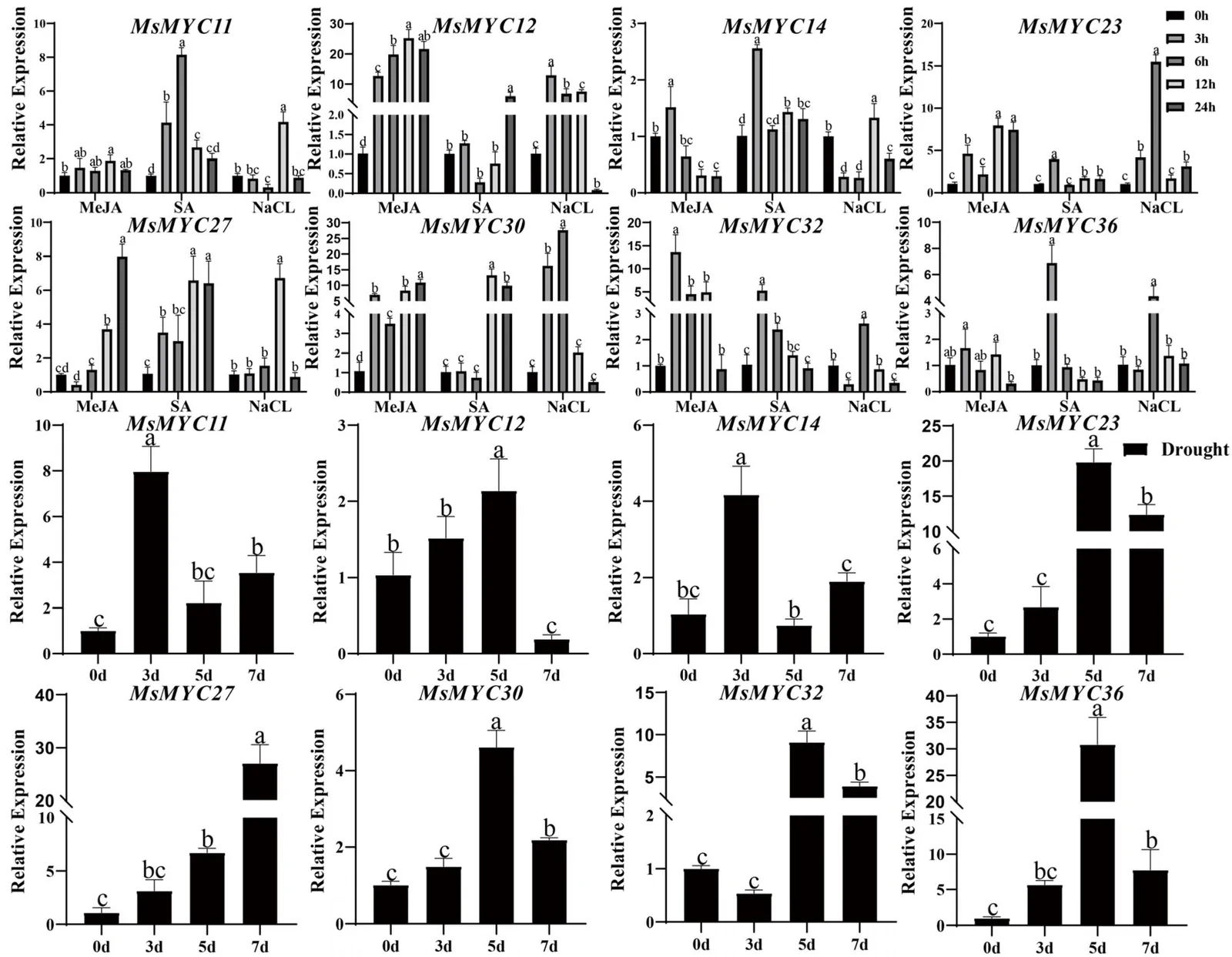
Expression patterns of eight selected *MsMYC* genes in alfalfa leaves under abiotic stress treatments. Columns correspond to the eight genes and rows to the four treatments (MeJA, SA, NaCl and drought). Each panel shows relative expression (y axis: 2^−ΔΔ^*^Ct^*, normalized to actin) against time (x axis: 0, 3, 6, 12 and 24 h for MeJA, SA and NaCl; 0, 3, 5 and 7 d for drought; the 0 h/0 d sample is the untreated control of the same treatment). Data are means +/− SD of three biological replicates (n = 3) and error bars are shown. Lowercase letters above the bars indicate significant differences among time points within a treatment (one-way ANOVA followed by Duncan’s multiple-range test, *p* < 0.05).

The results showed that under MeJA treatment, *MsMYC14* and *MsMYC32* exhibited a transient induction pattern, both being rapidly upregulated at 3 h and reaching peak levels, followed by a gradual decline (Figure 7). *MsMYC23* and *MsMYC*30 displayed a biphasic induction pattern, with upregulation at 3 h, a subsequent decrease, and maintenance of relatively high levels at 12 h and 24 h. *MsMYC27* showed a delayed induction pattern, peaking at 24 h. *MsMYC11* exhibited low expression levels at all time points without significant changes. *MsMYC12* exhibited a gradual induction pattern, being significantly upregulated from 3 h onward, reaching its highest level at 12 h.

Under SA treatment, *MsMYC*11, *MsMYC*23, and *MsMYC*36 exhibited similar expression patterns, all being rapidly upregulated and reaching peaks at 3 h or 6 h, respectively, followed by a gradual decline (Figure 7). *MsMYC*14 and *MsMYC32* showed a transient induction pattern, both peaking at 3 h. *MsMYC27* and *MsMYC30* displayed a delayed induction pattern, peaking at 12 h. *MsMYC12* was significantly suppressed at 6 h and then recovered significantly to a peak at 24 h.

Under NaCl treatment, the eight validated genes also showed distinct temporal expression profiles (Figure 7). *MsMYC12* was rapidly induced, reaching its highest level at 3 h and then declining gradually. *MsMYC23* also showed an early response but peaked later, reaching its maximum at 6 h and declining rapidly thereafter. *MsMYC27* showed a distinct late-peaking kinetic, with a sharp peak at 12 h, whereas its expression at the other time points remained low. The remaining genes showed transient or modest changes without a consistent temporal trend.

Under drought stress, *MsMYC*11 and *MsMYC*14 exhibited similar expression patterns, both being rapidly upregulated at 3 d and reaching peak levels, followed by a decline (Figure 7). *MsMYC*23, *MsMYC*30, *MsMYC*32, and *MsMYC*36 showed similar expression patterns, all remaining at relatively low levels from 0 to 3 d, then being significantly upregulated at 5 d and reaching peak levels, followed by a decline; among them, *MsMYC*23 and *MsMYC*36 showed particularly high peak levels. *MsMYC*12 displayed a delayed induction pattern, peaking at 5 d and rapidly declining to baseline at 7 d. *MsMYC*27 also showed a delayed induction pattern, reaching a significant peak at 7 d.

Overall, MeJA, SA, NaCl and drought treatments all modulated the expression of the eight selected *MsMYC* genes, with the largest changes observed under MeJA and drought. Among them, *MsMYC*12, *MsMYC*23, and *MsMYC*36 showed particularly strong responses, with expression upregulated by more than 5-fold under stresses. *MsMYC*23 exhibited an “relatively early” pattern under NaCl stress, being upregulated to a peak at 6 h and then rapidly declining to baseline, suggesting its potential involvement in the early transcriptional response to salt stress in alfalfa. *MsMYC*36 displayed a strong cumulative response pattern under drought stress, reaching the highest peak among the eight validated genes at 5 d, and although it declined at 7 d, it remained at a relatively high level, indicating that it is a candidate regulator of the late phase of the drought response in alfalfa. In addition, *MsMYC*12 exhibited a distinct antagonistic regulation pattern: it was rapidly and strongly induced under MeJA treatment, significantly suppressed from 0 to 12 h under SA treatment before recovering to a peak at 24 h, and showed high expression at all time points except 24 h under NaCl treatment. These kinetics make *MsMYC*12 a candidate for involvement in JA-SA-salt crosstalk and for contributing to the divergence and integration of different stress signals. *MsMYC*27 exhibited a “delayed response” characteristic under multiple stresses, peaking at 24 h under MeJA treatment, at 12 h under SA treatment, at 12 h under NaCl treatment, and at 7 d under drought stress, suggesting that this gene is potentially associated with late-phase stress responses in alfalfa, possibly participating in adaptive regulation or damage repair.

### 2.7. Subcellular Localization Analysis

**Figure 8:**
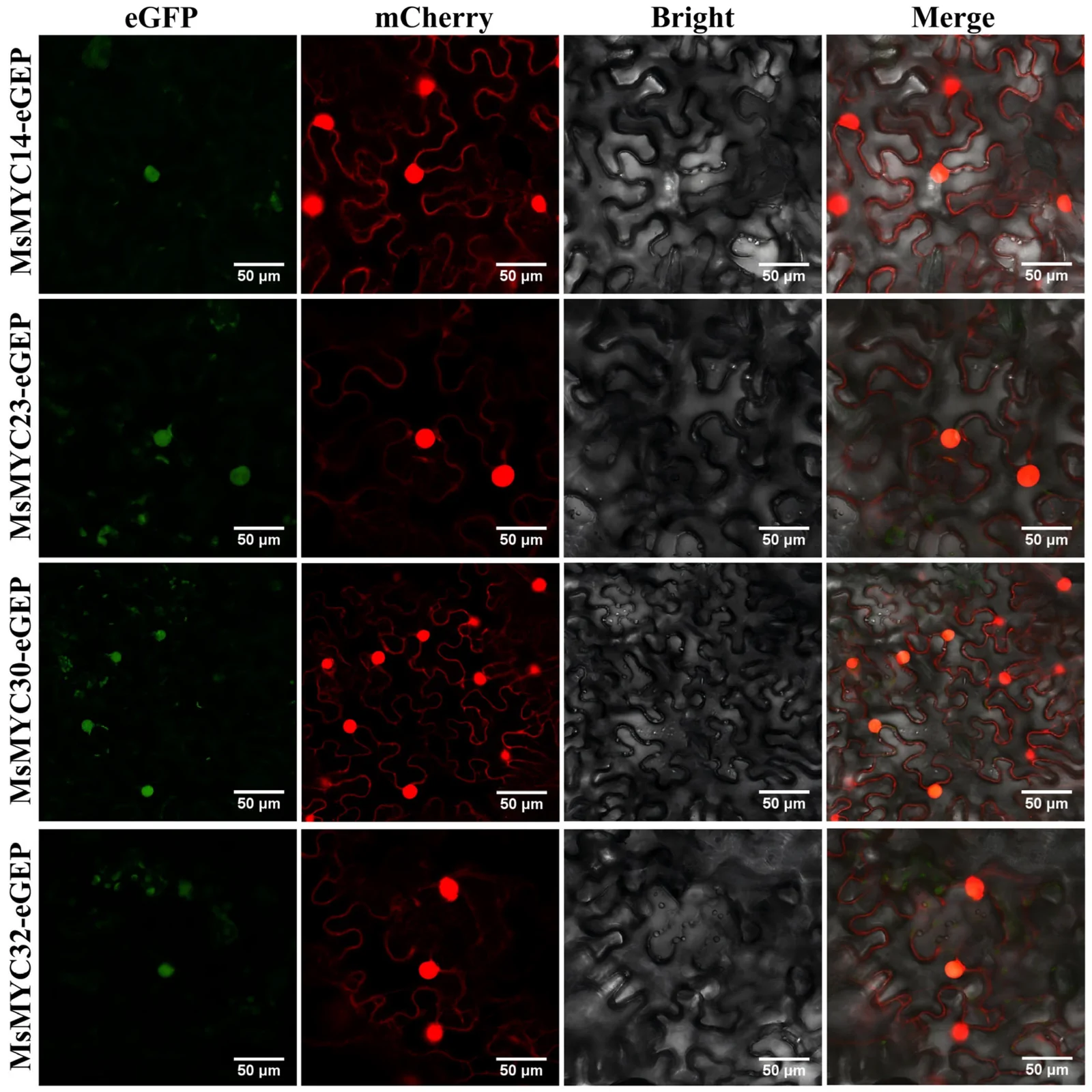
Subcellular localization of selected MsMYC proteins. Representative confocal images of Nicotiana benthamiana leaf epidermal cells transiently co-expressing MsMYC-eGFP fusions (green) and the nuclear marker (red). From left to right, the panels show the eGFP channel, the nuclear marker channel, the bright-field image, and the merged image of the same cell; the merged image indicates that the green signal co-localized with the nuclear marker. Scale bars: 50 μm. Images for the remaining four MsMYC proteins are provided in Figure S3.

As transcription factors, the regulatory function of MsMYC proteins fundamentally depends on their ability to enter the nucleus and bind to downstream target gene promoters. Given that the eight candidate genes identified in Section 2.6 exhibited distinct and specific expression patterns under abiotic stress, experimental analysis of their subcellular localization is essential to link their transcriptional activity to their potential physiological roles. Therefore, the eGFP fusion proteins of *MsMYC11*, *MsMYC12*, *MsMYC14*, *MsMYC23*, *MsMYC27*, *MsMYC30*, *MsMYC32* and *MsMYC36* were transiently expressed in *Nicotiana benthamiana* leaf epidermal cells together with the nuclear marker (Section 4.7). For all eight proteins, green fluorescence was restricted to the nucleus and co-localized with the red nuclear marker; representative images for four proteins are shown in Figure 8, and images for the remaining four proteins are provided in Figure S3. These results not only support the in silico prediction that all 38 MsMYC proteins are nuclear (Table S2), but more importantly, they provide spatial evidence that these eight stress-responsive candidates are structurally and subcellularly competent to act as nuclear transcriptional regulators in alfalfa.

## 3. Discussion

Alfalfa (*Medicago sativa* L.), as an autotetraploid legume forage, possesses a unique polyploid genome background that leads to significant differences in gene family evolution and stress regulatory networks compared with diploid model plants. *MYC* transcription factors, as core hubs in the bHLH family that regulate hormone signaling and abiotic stress responses, represent a key entry point for understanding the evolution of environmental adaptability in polyploid plants. However, systematic studies of this family in polyploid alfalfa remain lacking.

This study represents a genome-wide characterization of the *MYC* subfamily in autotetraploid alfalfa, identifying a total of 38 *MsMYC* sequences. To avoid counting homoeologous alleles as independent paralogs, these 38 sequences were collapsed into 12 ancestral loci based on allele-aware synteny (Table S10). This number is comparable to the 13 *MYC* genes reported in the diploid relative *Medicago truncatula*, whereas it is lower than that of the paleopolyploid *Glycine max* (43). To ensure a fair comparison, *MYC* genes in *M. truncatula* and *G. max* were re-identified using the same pipeline and thresholds applied to alfalfa, recovering 13 and 43 members, respectively. The counts for *Arabidopsis thaliana* (16) and *Oryza sativa* (7) were taken from previously published studies.

At the locus level, the apparent high number of *MsMYC* sequences (38) in autotetraploid alfalfa largely reflects the retention of multiple homoeologs per locus (up to four), rather than the birth of new paralogs. By contrast, the 43 *MYC* genes in the paleopolyploid *G. max* reflect true paralogous expansion after two rounds of WGD and subsequent diploidization. This distinction is critical: autotetraploidy increases allelic dosage and provides raw material for subfunctionalization among homoeologs, whereas paleopolyploidy generates new paralogous loci. Furthermore, the retention of homoeologous alleles in alfalfa was accompanied by an ancient segmental duplication and four tandem duplications (Section 2.4). Together, these duplication and retention events involved 27 distinct *MsMYC* sequences, accounting for 71.1% (27/38) of the family. Meanwhile, the *Ka*/*Ks* ratios of the 12 ancient segmental paralog pairs (*Ks* 0.74–0.79) were all significantly less than 1 (Fisher’s exact test, *p* < 0.0001), indicating that these paralogs have been under strong purifying selection. By contrast, the 34 recent WGD-derived homoeologous pairs (*Ks* ≤ 0.116) contain very few substitutions, and their *Ka*/*Ks* values reflect allelic variation rather than long-term selection. Despite this purifying selection on ancient paralogs, the increased copy number provides genetic material for subsequent functional divergence. As core transcription factors regulating growth, development, and stress responses, MYC proteins possess highly indispensable functions. Therefore, the increase in copy number caused by homoeolog retention and duplication does not lead to the loss of original functions; instead, purifying selection has maintained the integrity of the core regulatory network. This “duplication–retention–purification” pattern is consistent with the greater environmental plasticity of polyploid plants compared with diploids.

These evolutionary and expression features can be compared with those of other transcription factor families that have been characterized in alfalfa. Genome-wide surveys of the NAC, ARF, HSF, PLATZ and bZIP families, and more recently of the MADS-box family, have all reported expansion of the family in the tetraploid genome followed by functional divergence among duplicated copies [25]. The MYC family conforms to this general pattern (38 members and 46 WGD/segmental duplicated pairs), but two features appear distinctive. First, the retention rate of *MYC* copies relative to the diploid relative *M. truncatula* is high (71.1%), and homoeologous copies frequently show divergent temporal kinetics rather than coordinated expression (Section 2.6). Second, within the hormone-responsive class, jasmonate- and abscisic-acid-responsive elements were prominent in the promoters of *MYC* duplicates, whereas families such as *HSF* and *NAC* are enriched in heat- and dehydration-responsive elements, respectively [5,7]; overall, however, light-responsive elements were the most abundant (513), followed by hormone-responsive (250) and stress-responsive (147) elements (Section 2.5). These comparisons suggest that the MYC family contributes a hormone-centered layer to the stress regulatory network of polyploid alfalfa that is complementary to the more direct stress-sensing roles of other families.

Differences in cis-acting element composition provide clues to possible expression divergence within the MsMYC family at the transcriptional regulatory level. We therefore examined the temporal kinetics of eight selected members under salt, drought, and hormone treatments, which revealed three representative behaviors (Figure 7). Early peaking represented by *MsMYC23* (rapid activation at 6 h under salt stress followed by a rapid decline), late peaking represented by *MsMYC36* (a peak at 5 d under drought stress), and opposite-direction expression changes represented by *MsMYC12*. These expression patterns suggest that members with early-peaking kinetics may participate in early transcriptional reprogramming under salt stress, whereas members with late-peaking kinetics may be involved in adaptive regulation during prolonged drought. The accumulation of osmolytes such as proline and soluble sugars is an important mechanism of plant drought tolerance; the expression kinetics of *MsMYC36* are correlated with the temporal characteristics of these slow response processes. *MsMYC12* showed opposite-direction expression changes under MeJA and SA treatments. Previous studies have shown that JA and SA signaling often exhibit antagonistic relationships in plant defense responses, but their interaction patterns vary with stress type and intensity.

Because alfalfa is a recent autotetraploid rather than a paleopolyploid, the fate of *MYC* duplicates is expected to be governed by mechanisms specific to young polyploids. Three non-exclusive models can be invoked. First, dosage balance predicts that genes whose products act within stoichiometrically sensitive complexes, as transcription factors do, are preferentially retained after whole-genome duplication, because loss of a single copy perturbs the balance among interaction partners; this expectation is consistent with the high retention rate of *MYC* copies and with the uniformly low *Ka/Ks* values observed for duplicated pairs. Second, subfunctionalization predicts partitioning of the ancestral expression or functional repertoire among the retained copies; the finding that homoeologous members such as *MsMYC11* and *MsMYC12*, and the tandem pair *MsMYC23*/*MsMYC27*/*MsMYC30* region, differ markedly in their temporal kinetics under MeJA, SA, NaCl and drought treatments (Figure 7) provides expression-level evidence for this process. Third, neofunctionalization predicts the acquisition of qualitatively new functions, which cannot be assessed from expression data alone. On the available evidence, we therefore favor a combination of dosage-driven retention and subfunctionalization, with neofunctionalization remaining a hypothesis. Because tetraploid alfalfa is largely disomic and is still undergoing diploidization, the family may not yet have reached a stable post-polyploid equilibrium, and the biased-fractionation signature typical of older polyploids may not yet be detectable.

Subcellular localization results showed that all eight selected genes were localized to the nucleus, consistent with their roles as transcription factors. This provides a subcellular basis for their involvement in stress response regulation.

Several limitations of this study should be stated. First, expression analysis was restricted to leaves; because salt stress is perceived first by the root system and MYC proteins also function in root development and rhizobial symbiosis, the conclusions drawn here are limited to the leaf compartment. Second, the eight genes analyzed here were selected based on phylogenetic representation, promoter cis-element composition, and domain completeness, and they cannot be regarded as representative of the 38-member family; the temporal patterns described are therefore properties of this subset rather than of the family as a whole. Finally, subcellular localization was assessed only by transient expression in Nicotiana benthamiana, and only eight of the 38 members were examined.

In summary, NaCl, MeJA, SA, and drought treatments modulated the expression of most of the eight *MsMYC* genes examined, and the expression data suggest that *MsMYC12*, *MsMYC23* and *MsMYC36* may be associated with JA-SA crosstalk, the early salt response and the late drought response, respectively. Future work should test these hypotheses through expression correlation, target prediction, and functional analysis. This study lays a foundation for investigating the roles of target genes in abiotic stress responses and provides important candidate gene resources for breeding stress-tolerant forage crops.

## 4. Materials and Methods

### 4.1. Genome-Wide Identification and Characterization of the MsMYC Gene Family

The genome of alfalfa (*Medicago sativa* L. cv. ‘Xinjiang Daye’) was downloaded from the figshare project “Whole genome sequencing and assembly of Medicago sativa”(https://figshare.com/projects/whole_genome_sequencing_and_assembly_of_Medicago_sativa/66380, accessed on 1 April 2025); the corresponding allele-aware chromosome-level assembly, comprising 32 pseudochromosomes, was described by Chen et al. Genome coordinates and gene identifiers for all 38 *MsMYC* genes are listed in Table S1. Hidden Markov model (HMM) profiles of the conserved MYC domains were downloaded from the Pfam database (http://pfam.xfam.org, accessed on 1 April 2025), with PF14215 corresponding to the MYC_N/JID domain and PF00010 corresponding to the bHLH domain. In parallel, BLASTP searches were performed using *Arabidopsis thaliana* MYC protein sequences as queries against the alfalfa protein database with an E-value cutoff of 1 × 10^−5^. These HMM profiles were used to search against the alfalfa protein sequences using TBtools software (v2.326) with an E-value cutoff of 1 × 10^−5^ [26]. After the candidate sequences were obtained, the NCBI Batch CD-search (https://www.ncbi.nlm.nih.gov/cdd/, accessed on 1 April 2025), InterPro (http://www.ebi.ac.uk/interpro, accessed on 1 April 2025), and SMART (https://smart.embl.de/, accessed on 1 April 2025) databases [27,28] were used to verify whether these candidate sequences contained conserved MYC domains. After verification, the members of the *MYC* gene family in alfalfa were obtained. The physicochemical properties of the MsMYC family members, including amino acid length, molecular weight, and isoelectric point, were analyzed using the ExPASy online server (http://www.expasy.org, accessed on 1 April 2025) [29]. Subcellular localization of the MsMYC family was predicted using CELLO (http://cello.life.nctu.edu.tw, accessed on 1 April 2025) and WoLF PSORT (https://www.genscript.com/wolf-psort.html, accessed on 1 April 2025) with default parameters. Based on the reference genome annotation of alfalfa, chromosomal positions were visualized using the Gene Location Visualize function of the GTF/GFF tool in TBtools (v2.326) [30]. The identified MYC family members were subsequently named according to their positions on the chromosomes.

To compare the family size under identical criteria, *MYC* genes in *M. truncatula* and *G. max* were re-identified using the same BLASTP and HMMER pipeline and thresholds described above. The proteomes of these two species were downloaded from Ensembl Plants. Candidate sequences containing both the MYC_N and bHLH domains were retained, yielding 13 and 43 members in *M. truncatula* and *G. max*, respectively.

### 4.2. Phylogenetic and Multiple Sequence Alignment Analysis of MsMYC Proteins

MYC protein sequences from *Arabidopsis* and other plant species were obtained from TAIR and NCBI (https://www.ncbi.nlm.nih.gov/, accessed on 10 April 2025). Phylogenetic trees of MYC protein sequences from *Medicago sativa*, *Arabidopsis thaliana*, *Medicago truncatula*, *Glycine max* and *Oryza sativa* were constructed using MEGA 11 software with the neighbor-joining (NJ; p-distance model, pairwise deletion of gaps) and maximum likelihood (ML; LG + F + R model, selected from 56 substitution models by the corrected Akaike information criterion) methods (Figure S4), with 1000 bootstrap replicates [31]. The resulting NWK files were visualized using the iTOL website (https://itol.embl.de, accessed on 10 April 2025) [32]. Multiple sequence alignment of MsMYC family protein sequences was performed using the ClustalW algorithm integrated in MEGA 11 [33]. All parameters were kept at the default settings for protein alignment: the Gonnet protein weight matrix was used for amino acid substitution scoring; gap penalties were applied using the affine gap penalty model, with a gap opening penalty of 10 and a gap extension penalty of 0.2 [34]. The delay divergent cutoff was set to 30%. The alignment results were manually inspected, and conserved MYC domain regions were extracted for subsequent phylogenetic analysis.

### 4.3. Conserved Motifs, Domains, and Gene Structure of the MsMYC Gene Family

The motif composition of the *MsMYC* gene family was analyzed using the MEME online tool (https://meme-suite.org/meme/tools/meme, accessed on 10 April 2025) with the number of motifs set to 10 and all other parameters kept at default settings [35]. Conserved domains were predicted using the NCBI CDD database (https://www.ncbi.nlm.nih.gov/cdd/, accessed on 10 April 2025). Conserved motifs, domains, and gene structures were visualized using the Gene Structure View (Advanced) tool in TBtools (v2.326).

To examine the coding potential and exclude potential retrocopies or annotation artifacts, the coding sequences (CDS) of all 38 *MsMYC* genes were extracted. The open reading frame (ORF) integrity was evaluated using the EMBOSS getorf tool (with a minimum ORF size of 75 nt, searching for regions between START and STOP codons). Genes were classified as “Complete” if they contained a start codon, a terminal stop codon, and no internal premature stop codons, or “Partial” if they contained internal stop codons. The exon number for each gene was determined from the GFF3 annotation file and cross-validated with the gene structure visualized in TBtools.

### 4.4. Identification of Gene Duplication Events and Collinearity in the MsMYC Gene Family

The genome and GFF files of *Arabidopsis thaliana*, *Medicago truncatula*, *Glycine max*, and *Oryza sativa* were downloaded from the Ensembl Plants database (https://plants.ensembl.org/, accessed on 10 April 2025); the alfalfa genome and GFF files were obtained as described in Section 4.1. Intraspecific collinearity within alfalfa and interspecific collinearity between alfalfa and other species were analyzed in TBtools (v2.326) with an E-value threshold of 1 × 10^−5^, a minimum block size of 5, and a maximum gap of 25 genes. Tandem duplicates were defined as family members located on the same chromosome within 100 kb of one another and separated by no more than 10 intervening genes, and were identified with the Duplicate Gene Classifier of MCScanX. After excluding tandem duplicates, WGD/segmental duplicates were defined as those retained from the intra-species collinearity analysis. To distinguish recent WGD-derived homoeologous pairs from older paralogous duplicates, each pair was first assigned to homologous groups and homoeologous chromosomes based on the allele-aware annotation, and its syntenic topology was inspected. A pair was classified as a recent WGD-derived homoeologous pair when both loci mapped to different homoeologous chromosomes within the same homologous group and were embedded in the same four-way homoeologous syntenic block; *Ks* was used only as supporting evidence, not as a stand-alone criterion. *Ka* and *Ks* were calculated with the *KaKs*_Calculator module in TBtools using the Yang–Nielsen (YN) model. Pairs with *Ks* > 2 were excluded from the selection analysis because synonymous substitutions are saturated in such pairs, and pairs with *Ks* = 0 were also excluded because *Ka*/*Ks* is undefined. *Ka*/*Ks* < 1 was taken to indicate purifying selection. The statistical significance of the *Ka*/*Ks* ratios was evaluated using Fisher’s exact test provided by *KaKs*_Calculator, with *p* < 0.05 considered significant.

### 4.5. Promoter Cis-Acting Regulatory Element Analysis of the MsMYC Gene Family

The 2 kb sequences upstream of the start codon of *MsMYC* genes were extracted using TBtools software (v2.326). These sequences were then submitted to the PlantCARE online website (https://ngdc.cncb.ac.cn/databasecommons/database/id/5261, accessed on 10 April 2025) for prediction of cis-acting elements in the promoter regions [36]. Visualization and heatmap analysis of cis-acting elements were performed using the Heatmap tool in TBtools (v2.326). In addition, the number and category of each type of cis-acting element were counted and summarized, and the results were presented as stacked bar charts generated using GraphPad Prism software (v10.1.2). The general core promoter elements (TATA-box and CAAT-box) were excluded from the functional interpretation. Promoter cis-element composition was summarized descriptively for each gene and for the family as a whole.

### 4.6. Abiotic Stress Treatments and qRT-PCR Analysis

In this study, alfalfa (*Medicago sativa* L. cv. ‘Xinjiang Daye’) was used as the plant material. The alfalfa seeds used in this study were self-saved seeds maintained in our laboratory. Alfalfa seeds were sown in nutrient soil and cultivated at 25 °C under a 16 h light/8 h dark photoperiod for three weeks. Seedlings were then transferred to hydroponic containers containing half-strength Hoagland nutrient solution and grown for an additional week. Uniformly growing plants were selected and subjected to NaCl, MeJA, SA, and drought treatments, respectively. The treatment methods were as follows: 4-week-old seedlings were treated with salt (200 mmol L^−1^ NaCl), MeJA (25 μmol L^−1^) and SA (100 μmol L^−1^) for 24 h, and with drought for 7 d. NaCl was added to the hydroponic solution to reach the final concentration indicated. MeJA and SA were applied by foliar spraying of the whole shoot until run-off. For the time-course qRT-PCR analysis, the 0 h (or 0 d) sample served as the single baseline, and relative expression was calculated exclusively against this baseline. For the salt and hormone treatments, leaves were collected at 0, 3, 6, 12 and 24 h (0 h served as the untreated control), and each treatment included three biological replicates. For drought treatment, the pot water control method was used to simulate natural drought stress. Uniformly growing plants were transplanted into pots containing an equal volume of mixed substrate (nutrient soil:perlite:vermiculite = 3:1:1). After two weeks of recovery, the plants were divided into a control group and a drought treatment group. Before treatment, all pots were watered thoroughly to saturate the soil moisture content. From 0 to 7 d, watering was completely stopped for the drought treatment group, while the control group was watered normally. Functional leaves from the same position were collected at 0, 3, 5, and 7 d after water withholding. Three biological replicates were included for each treatment at each time point. All collected samples were immediately frozen in liquid nitrogen for total RNA extraction. Total RNA was extracted from frozen leaf samples using the FastPure Universal Plant Total RNA Isolation Kit (Vazyme, Nanjing, China) according to the manufacturer’s instructions. RNA quality was assessed by agarose gel electrophoresis. Residual genomic DNA removal and first-strand cDNA synthesis were performed using the FastKing One-Step gDNA Removal and cDNA Synthesis Premix (Tiangen, Beijing, China) according to the manufacturer’s instructions. qRT-PCR was performed on the Roche LightCycler^®^ 480 system (Roche, Basel, Switzerland) using Vazyme SYBR reagents (ChamQ Universal SYBR qPCR Master Mix, Vazyme, Nanjing, China). Each 20 μL reaction contained 10 μL of 2× ChamQ Universal SYBR qPCR Master Mix, 0.6 μL of each gene-specific primer, 1.3 μL of diluted cDNA, and 7.5 μL of sterile water. The amplification protocol consisted of an initial pre-incubation at 95 °C, followed by 45 cycles of 95 °C, 57 °C and 72 °C, and a melting-curve analysis from 60 °C to 95 °C.

Raw Ct values were acquired using the Roche LightCycler^®^ 480 software with default baseline and threshold settings. The specificity of each primer pair was verified by the single peak in the melting-curve analysis. For each biological replicate, the three technical replicates were directly averaged to obtain the mean Ct value used for subsequent calculations. Gene-specific primers were designed using Primer Premier 6.0 software (San Francisco, USA; https://premierbiosoft.com, accessed on 10 April 2025) for qRT-PCR. The alfalfa actin gene was used as the internal reference gene [25]. All primers used in this study are listed in Table S5. The relative expression levels were calculated using the 2^−ΔΔ*Ct*^ method proposed by Livak and Schmittgen [37]. For each treatment and time point, leaf samples were collected from independent biological replicates (different individual plants). For data analysis, the three technical replicates of each biological sample were averaged prior to calculating the relative expression, with the 0 h (or 0 d) sample as the untreated baseline. One-way ANOVA followed by Duncan’s multiple-range test was performed on the mean relative expression values of the three biological replicates (n = 3) to compare all time points within a treatment (all pairwise comparisons, including the 0 h baseline). *p* < 0.05 was considered statistically significant, and *p* < 0.01 highly significant.

### 4.7. Subcellular Localization Assay

The full-length coding sequences of the eight *MsMYC* genes were cloned into the pCAMBIA1300-eGFP vector stored in our laboratory, which had been digested with BamHI and SalI (TaKaRa, Dalian, China), such that eGFP was fused to the C-terminus of each MsMYC protein (MsMYC-eGFP); all fusion junctions were verified by Sanger sequencing. The constructs were ligated using a homologous recombination kit (Vazyme, Nanjing, China) and the resulting products were transformed into *Escherichia coli* DH5α competent cells (preserved in our laboratory). Positive clones confirmed by Sanger sequencing were further transformed into *Agrobacterium tumefaciens* strain GV3101 (preserved in our laboratory). Similarly, the nuclear marker plasmid pCAMBIA1300-35S-mCherry-NLS (Puint, Xianyang, China), which expresses a nuclear-localized mCherry fusion, was transformed into the same strain. The *Agrobacterium tumefaciens* cells carrying the recombinant plasmids were cultured in LB liquid medium containing kanamycin (50 mg/L) and rifampicin (25 mg/L) (Solarbio, Beijing, China) at 28 °C with shaking at 200 rpm for 24 h, and then the bacterial cells were collected. An infiltration solution containing 10 mmol L^−1^ MES, 10 mmol L^−1^ MgCl_2_ (pH 5.6), and 200 μM acetosyringone (AS) was prepared. The cultured bacterial suspension was added to the infiltration solution, and the OD_600_ was adjusted to 0.6–0.8. After incubation in the dark at room temperature for approximately 3 h, the mixture was injected into leaves of *Nicotiana benthamiana*, and the plants were kept in the dark for 2 d. Fluorescence signals were observed using a confocal laser scanning microscope (Nikon, Tokyo, Japan) at excitation wavelengths of 488 nm and 560 nm, respectively [38].

## 5. Conclusions

This study provides a genome-wide characterization of the *MYC* gene family in autotetraploid alfalfa. Thirty-eight *MsMYC* genes were identified and were unevenly distributed across 17 of the 32 pseudochromosomes, with 42.1% of them located in homologous group 8. These 38 genes collapsed into 12 ancestral loci based on allele-aware synteny, indicating that the high sequence number primarily reflects the retention of homoeologous alleles rather than true paralogous expansion. In addition to homoeolog retention, one ancient segmental duplication and four tandem duplications contributed to family expansion, and purifying selection (*Ka*/*Ks* < 1 for the 12 ancient segmental paralog pairs) has since preserved the core regulatory architecture of the family. Notably, eight representative members exhibit divergent temporal expression under salt, drought, MeJA and SA treatments, with *MsMYC12*, *MsMYC23* and *MsMYC36* showing the most pronounced changes; all eight proteins localized to the nucleus in a transient assay. The main implication of these results is that polyploid alfalfa has retained a large and partially divergent set of *MYC* genes whose members are likely to contribute to different phases of the stress response and to the crosstalk between jasmonate and salicylate signaling. Functional analysis of these candidates is a priority for future work.

## Data Availability

The original contributions presented in this study are included in the article/Supplementary Material. Further inquiries can be directed to the corresponding author(s).

## Supplementary Materials

The following supporting information can be downloaded at: https://www.mdpi.com/article/10.3390/ijms27219964/s1.

## References

[1] Desta A.G. (2026). Medicago sativa for climate-smart agriculture and soil sustainability: A review. Discov. Environ..

[2] Fang Y., Jiang G., Du P., Wang J., He H., Li H., Li H., Wang F., Xie Q. (2025). Genome-Wide Identification and Functional Divergence of the Chloride Channel (CLC) Gene Family in Autotetraploid Alfalfa (*Medicago sativa* L.). Int. J. Mol. Sci..

[3] Wu D., Ma F., Ding F., Leng X., Hu Z., Wang H. (2026). Coupled Drip Irrigation and Microbial Inoculants Enhancing Alfalfa Yield and Optimizing Rhizosphere Fungal Communities in Arid Saline Soils. Agronomy.

[4] Evelin H., Devi T.S., Gupta S., Kapoor R. (2019). Mitigation of Salinity Stress in Plants by Arbuscular Mycorrhizal Symbiosis: Current Understanding and New Challenges. Front. Plant Sci..

[5] He F., Zhang L., Zhao G., Kang J., Long R., Li M., Yang Q., Chen L. (2022). Genome-Wide Identification and Expression Analysis of the NAC Gene Family in Alfalfa Revealed Its Potential Roles in Response to Multiple Abiotic Stresses. Int. J. Mol. Sci..

[6] Chen F., Zhang J., Ha X., Ma H. (2023). Genome-wide identification and expression analysis of the Auxin-Response factor (ARF) gene family in *Medicago sativa* under abiotic stress. BMC Genom..

[7] Ma J., Zhang G., Ye Y., Shang L., Hong S., Ma Q., Zhao Y., Gu C. (2022). Genome-Wide Identification and Expression Analysis of HSF Transcription Factors in Alfalfa (*Medicago sativa*) under Abiotic Stress. Plants.

[8] Li X., He F., Zhao G., Li M., Long R., Kang J., Yang Q., Chen L. (2023). Genome-Wide Identification and Phylogenetic and Expression Analyses of the PLATZ Gene Family in *Medicago sativa* L.. Int. J. Mol. Sci..

[9] Liu S., Qin B., Fang Q., Zhang W.-j., Zhang Z., Liu Y.-c., Li W.-j., Du C., Liu X., Zhang Y.-l. (2021). Genome-wide identification, phylogeny and expression analysis of the bZIP gene family in Alfalfa (*Medicago sativa*). Biotechnol. Biotechnol. Equip..

[10] Pires N., Dolan L. (2010). Origin and diversification of basic-helix-loop-helix proteins in plants. Mol. Biol. Evol..

[11] Kazan K., Manners J.M. (2013). MYC2: The master in action. Mol. Plant.

[12] Xu Y.H., Liao Y.C., Lv F.F., Zhang Z., Sun P.W., Gao Z.H., Hu K.P., Sui C., Jin Y., Wei J.H. (2017). Transcription Factor *AsMYC2* Controls the Jasmonate-Responsive Expression of *ASS*1 Regulating Sesquiterpene Biosynthesis in *Aquilaria sinensis* (Lour.) Gilg. Plant Cell Physiol..

[13] Gasperini D., Chételat A., Acosta I.F., Goossens J., Pauwels L., Goossens A., Dreos R., Alfonso E., Farmer E.E. (2015). Multilayered Organization of Jasmonate signaling in the Regulation of Root Growth. PLoS Genet..

[14] Qi T., Huang H., Song S., Xie D. (2015). Regulation of Jasmonate-Mediated Stamen Development and Seed Production by a bHLH-MYB Complex in Arabidopsis. Plant Cell.

[15] Qi T., Wang J., Huang H., Liu B., Gao H., Liu Y., Song S., Xie D. (2015). Regulation of Jasmonate-Induced Leaf Senescence by Antagonism between bHLH Subgroup IIIe and IIId Factors in Arabidopsis. Plant Cell.

[16] Guo D., Li J., Liu P., Wang Y., Cao N., Fang X., Wang T., Dong J. (2024). The jasmonate pathway promotes nodule symbiosis and suppresses host plant defense in *Medicago truncatula*. Mol. Plant.

[17] Abe H., Yamaguchi-Shinozaki K., Urao T., Iwasaki T., Hosokawa D., Shinozaki K. (1997). Role of arabidopsis *MYC* and *MYB* homologs in drought- and abscisic acid-regulated gene expression. Plant Cell.

[18] Shinozaki K., Yamaguchi-Shinozaki K. (2007). Gene networks involved in drought stress response and tolerance. J. Exp. Bot..

[19] Ohta M., Sato A., Renhu N., Yamamoto T., Oka N., Zhu J.K., Tada Y., Suzaki T., Miura K. (2018). MYC-type transcription factors, *MYC67* and *MYC70*, interact with *ICE1* and negatively regulate cold tolerance in *Arabidopsis*. Sci. Rep..

[20] Chini A., Fonseca S., Fernández G., Adie B., Chico J.M., Lorenzo O., García-Casado G., López-Vidriero I., Lozano F.M., Ponce M.R. (2007). The JAZ family of repressors is the missing link in jasmonate signaling. Nature.

[21] Figueroa P., Browse J. (2015). Male sterility in *Arabidopsis* induced by overexpression of a MYC5-SRDX chimeric repressor. Plant J..

[22] Lorenzo O., Chico J.M., Sánchez-Serrano J.J., Solano R. (2004). *JASMONATE-INSENSITIVE1* encodes a *MYC* transcription factor essential to discriminate between different jasmonate-regulated defense responses in *Arabidopsis*. Plant Cell.

[23] Liu J., Yang D., Li X., Jin Z., Li J. (2024). Deciphering the effect mechanism of chromosome doubling on the biomass increase in *Elsholtzia splendens*. Sci. Hortic..

[24] Wang L., Li Y., Li H., Li S., Song J., Guan W. (2025). Comparison of the morphological and physiological characteristics of diploid and tetraploid *Luculia pinceana* Hook. BMC Plant Biol..

[25] Dong X., Deng H., Ma W., Zhou Q., Liu Z. (2021). Genome-wide identification of the MADS-box transcription factor family in autotetraploid cultivated alfalfa (*Medicago sativa* L.) and expression analysis under abiotic stress. BMC Genom..

[26] Mistry J., Chuguransky S., Williams L., Qureshi M., Salazar G.A., Sonnhammer E.L.L., Tosatto S.C.E., Paladin L., Raj S., Richardson L.J. (2021). Pfam: The protein families database in 2021. Nucleic Acids Res..

[27] Potter S.C., Luciani A., Eddy S.R., Park Y., Lopez R., Finn R.D. (2018). HMMER web server: 2018 update. Nucleic Acids Res..

[28] Wang J., Chitsaz F., Derbyshire M.K., Gonzales N.R., Gwadz M., Lu S., Marchler G.H., Song J.S., Thanki N., Yamashita R.A. (2023). The conserved domain database in 2023. Nucleic Acids Res..

[29] Wilkins M.R., Gasteiger E., Bairoch A., Sanchez J.C., Williams K.L., Appel R.D., Hochstrasser D.F. (1999). Protein identification and analysis tools in the ExPASy server. Methods Mol. Biol..

[30] Chen C., Chen H., Zhang Y., Thomas H.R., Frank M.H., He Y., Xia R. (2020). TBtools: An Integrative Toolkit Developed for Interactive Analyses of Big Biological Data. Mol. Plant.

[31] Tamura K., Stecher G., Kumar S. (2021). MEGA11: Molecular Evolutionary Genetics Analysis Version 11. Mol. Biol. Evol..

[32] Letunic I., Bork P. (2024). Interactive Tree of Life (iTOL) v6: Recent updates to the phylogenetic tree display and annotation tool. Nucleic Acids Res..

[33] Larkin M.A., Blackshields G., Brown N.P., Chenna R., McGettigan P.A., McWilliam H., Valentin F., Wallace I.M., Wilm A., Lopez R. (2007). Clustal W and Clustal X version 2.0. Bioinformatics.

[34] Thompson J.D., Higgins D.G., Gibson T.J. (1994). CLUSTAL W: Improving the sensitivity of progressive multiple sequence alignment through sequence weighting, position-specific gap penalties and weight matrix choice. Nucleic Acids Res..

[35] Bailey T.L., Elkan C. (1994). Fitting a mixture model by expectation maximization to discover motifs in biopolymers. Proc. Int. Conf. Intell. Syst. Mol. Biol..

[36] Lescot M., Déhais P., Thijs G., Marchal K., Moreau Y., Van de Peer Y., Rouzé P., Rombauts S. (2002). PlantCARE, a database of plant cis-acting regulatory elements and a portal to tools for in silico analysis of promoter sequences. Nucleic Acids Res..

[37] Livak K.J., Schmittgen T.D. (2001). Analysis of relative gene expression data using real-time quantitative PCR and the 2(-Delta Delta C(T)) Method. Methods.

[38] Chen H., Zeng Y., Yang Y., Huang L., Tang B., Zhang H., Hao F., Liu W., Li Y., Liu Y. (2020). Allele-aware chromosome-level genome assembly and efficient transgene-free genome editing for the autotetraploid cultivated alfalfa. Nat. Commun..

