# Supplementary material for: Genome-Wide Identification, Characterization, and Expression Profiling of the MYC Gene Family in Autotetraploid Alfalfa (Medicago sativa L.)
Source: Int J Mol Sci. 2026 Nov 6;27(21):9964. doi: 10.3390/ijms27219964 (PMC13763828; doi:10.3390/ijms27219964)
Supplement: Supplementary file 1 [file ijms-27-09964-s001.zip › Supplementry Figures.pdf]

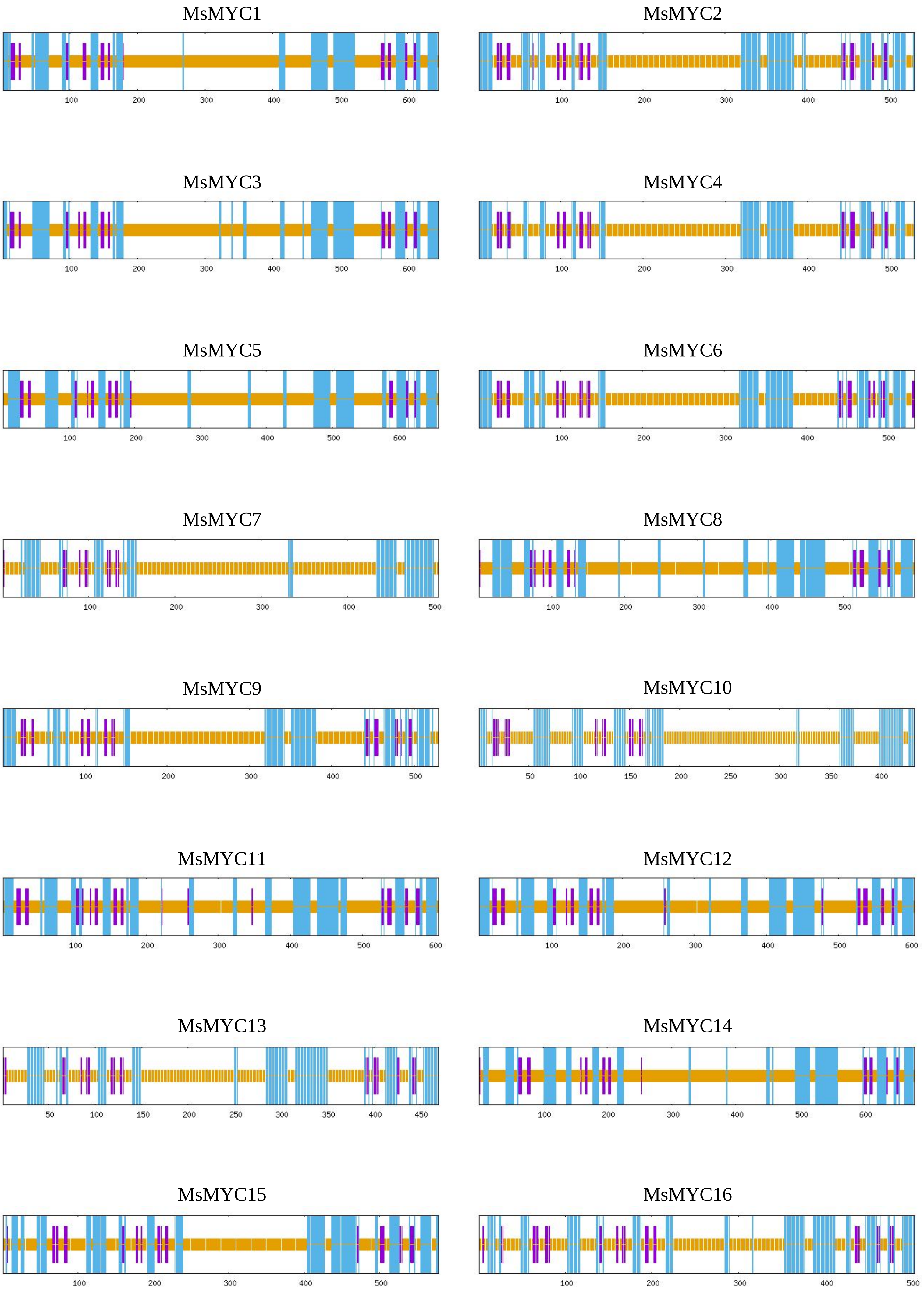
MsMYC1
100 200 300 400 500 600
MsMYC2
100 200 300 400 500
MsMYC3
100 200 300 400 500 600
MsMYC4
100 200 300 400 500
MsMYC5
100 200 300 400 500 600
MsMYC6
100 200 300 400 500
MsMYC7
100 200 300 400 500
MsMYC8
100 200 300 400 500
MsMYC9
100 200 300 400 500
MsMYC10
50 100 150 200 250 300 350 400
MsMYC11
100 200 300 400 500 600
MsMYC12
100 200 300 400 500 600
MsMYC13
50 100 150 200 250 300 350 400 450
MsMYC14
100 200 300 400 500 600
MsMYC15
100 200 300 400 500
MsMYC16
100 200 300 400 500

MsMYC17

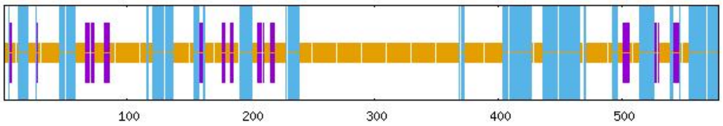

MsMYC18

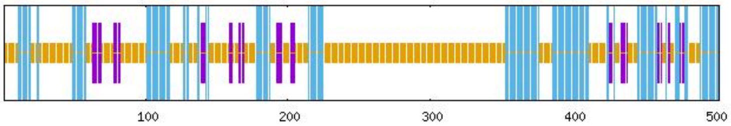

MsMYC19

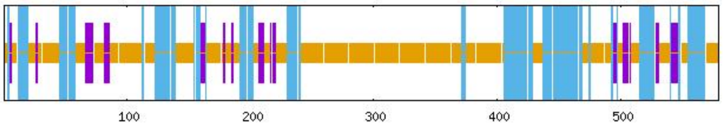

MsMYC20

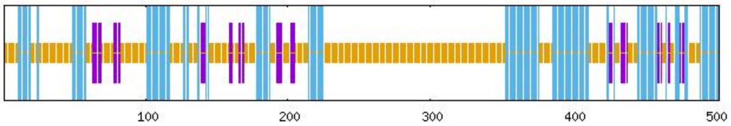

MsMYC21

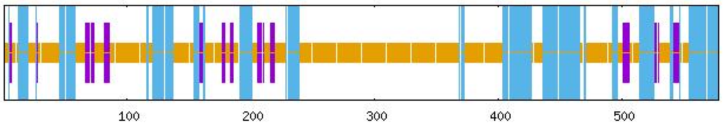

MsMYC22

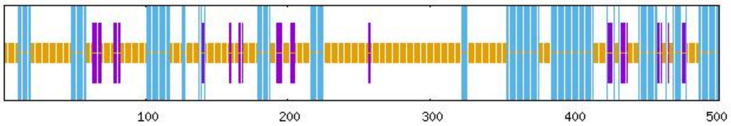

MsMYC23

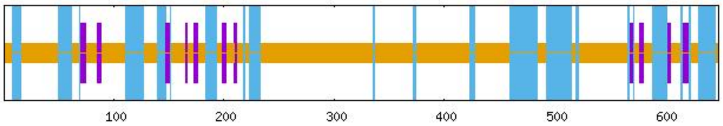

MsMYC24

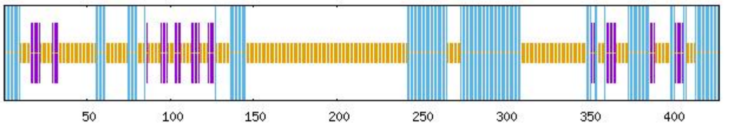

MsMYC25

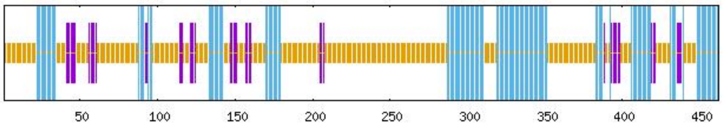

MsMYC26

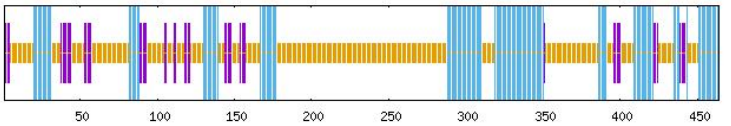

MsMYC27

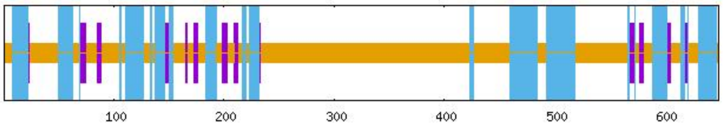

MsMYC28

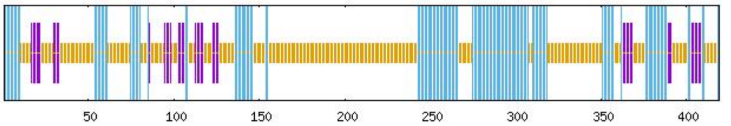

MsMYC29

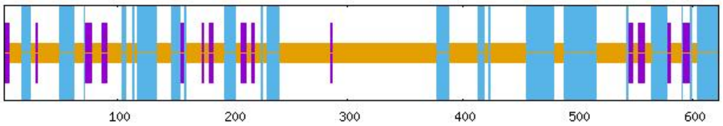

MsMYC30

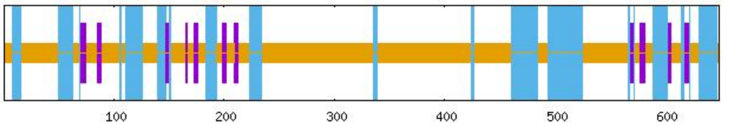

MsMYC31

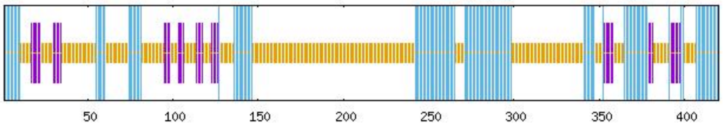

MsMYC32

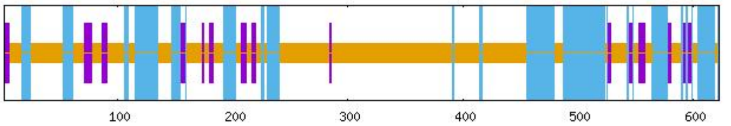

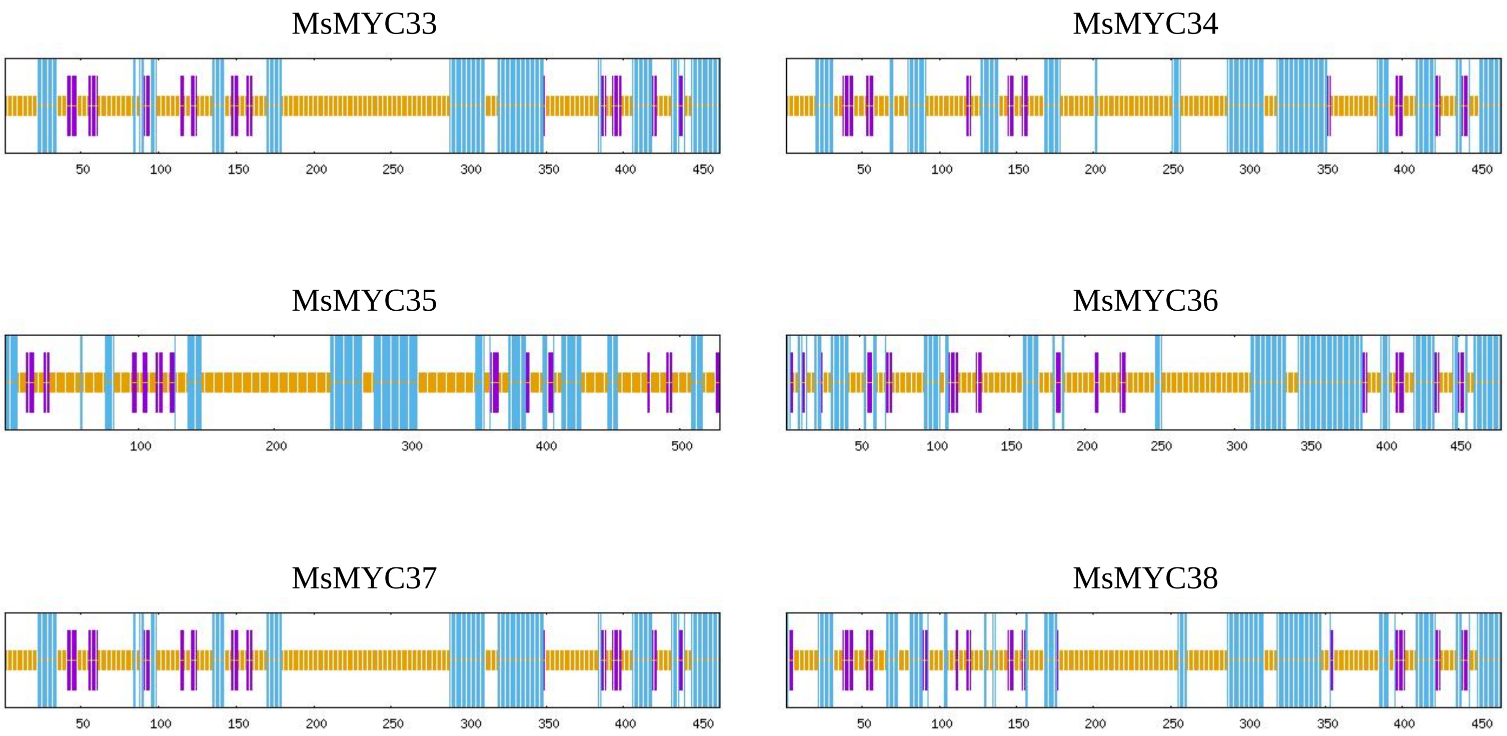

Figure S1. This is the prediction of the secondary structure of alfalfa MsMYCs, the colored part is the location of the corresponding secondary structure, blue represents α helix, purple represents extended chain, and yellow represents irregular curling.

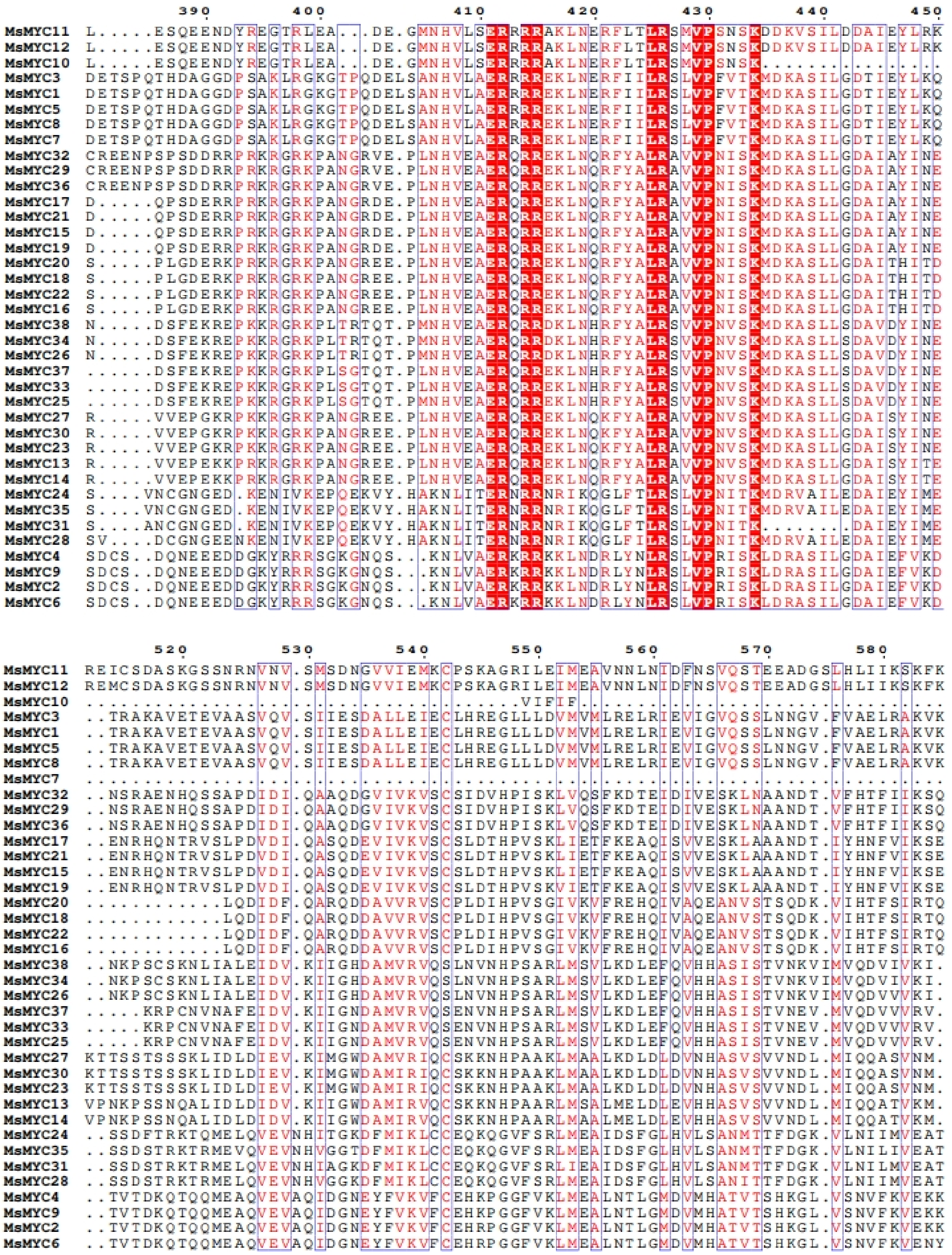

Figure S2 . Multiple sequence alignment of the MsMYCs family amino acid sequences. Amino acid residues marked in different colors represent their degree of conservation.

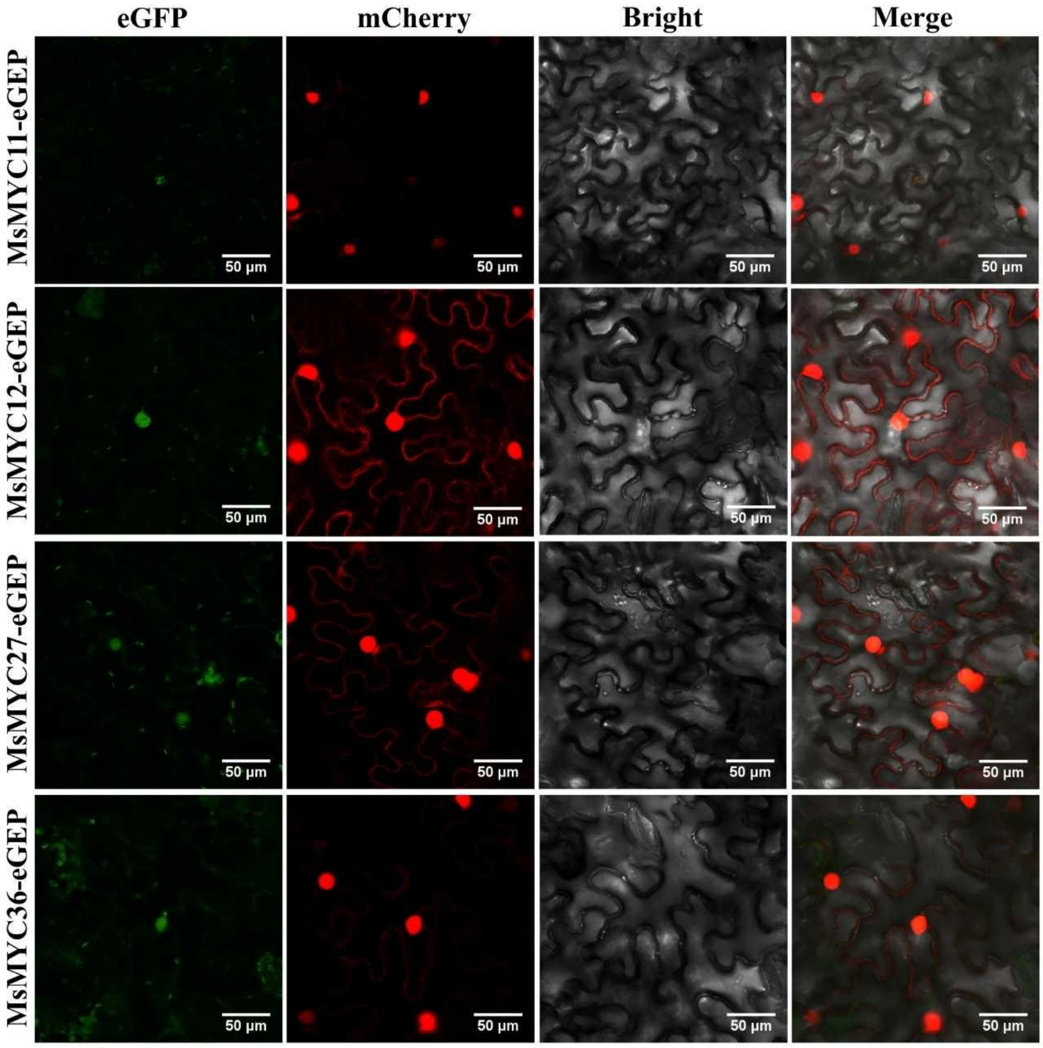

Figure S3 . Subcellular localization of the remaining four MsMYC proteins in Nicotiana benthamiana leaf epidermal cells. Representative confocal images of N. benthamiana leaf epidermal cells transiently co-expressing an MsMYC-eGFP fusion (green) and a nuclear marker(red). From left to right, the panels show the GFP channel, the nuclear marker channel, the bright-field image, and the merged image of the same cell; the merged image indicates that the green signal co-localizes with the nuclear marker. Scale bars, 50 µm. Each row represents one MsMYC protein. Similar nuclear localization patterns were observed for all four MsMYC proteins.

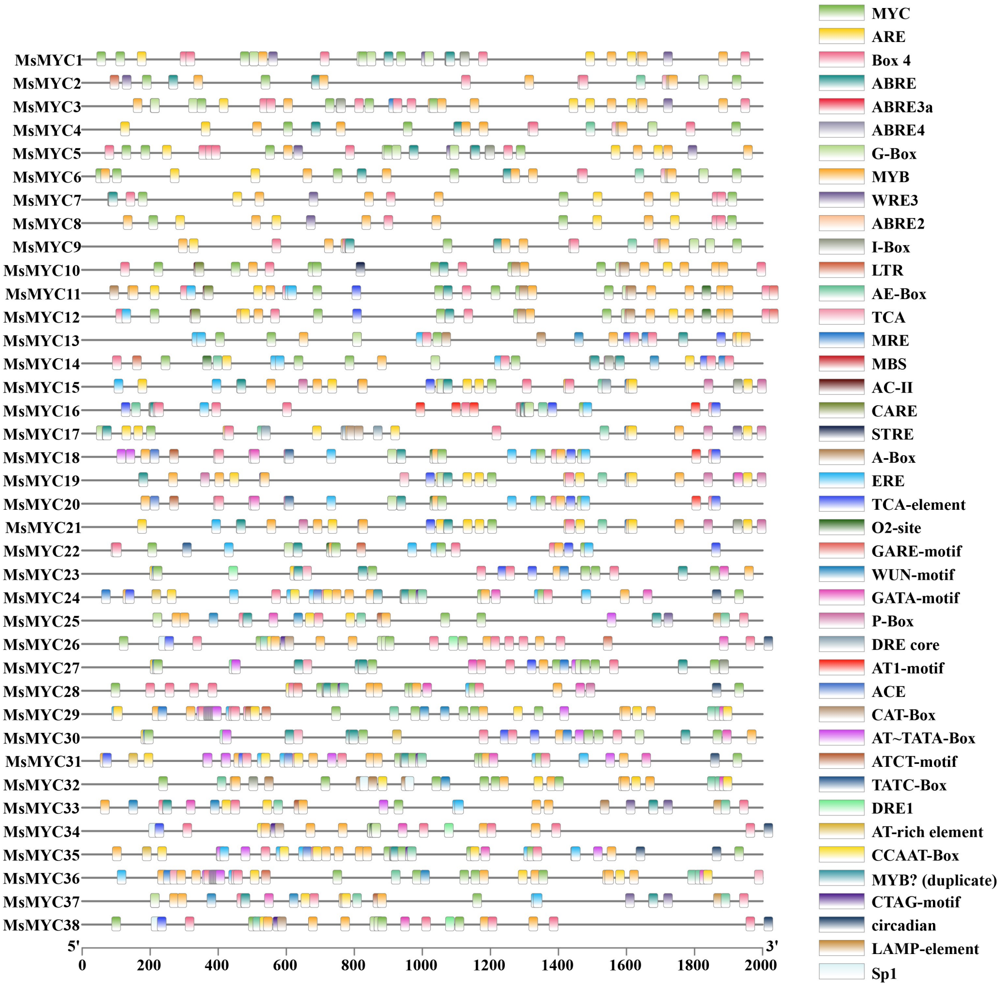

Figure S4 . Distribution of cis-acting regulatory elements in the promoters of the 38 *MsMYC* genes. The 2.0-kb sequences upstream of the start codon of *MsMYC1* – *MsMYC38* were analyzed using PlantCARE. Different colored boxes represent distinct motifs (ABRE, MYC, MYB, Box 4, G-Box, etc.), and their positions are shown relative to the start codon. The scale bar indicates nucleotide positions. Gene IDs are shown on the left.

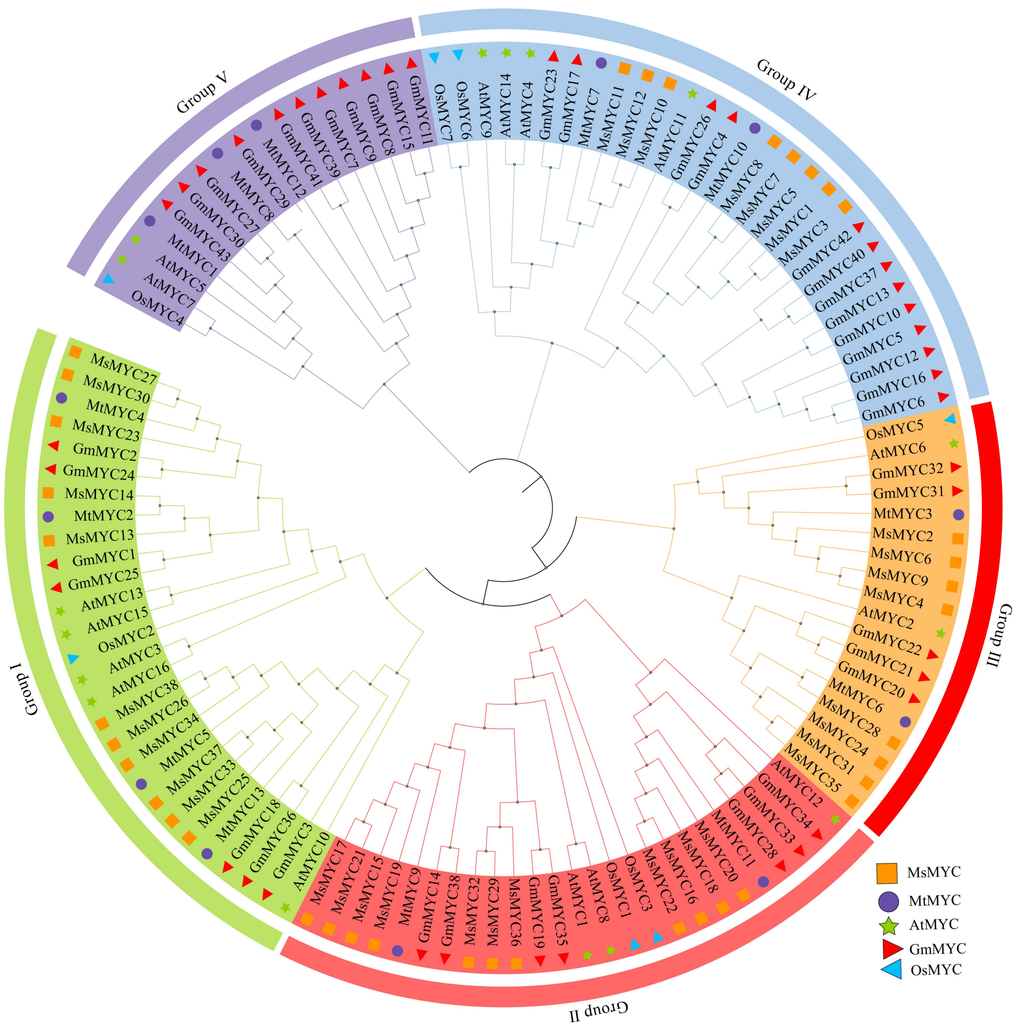

Figure S5 . Constructed to validate the topological stability of the Neighbor-Joining (NJ) tree. Bootstrap support values (≥50%) from 1,000 replicates are indicated at each node.
